# Sensor Fault-Tolerant Control of Microgrid Using Robust Sliding-Mode Observer

**DOI:** 10.3390/s22072524

**Published:** 2022-03-25

**Authors:** Ebrahim Shahzad, Adnan Umar Khan, Muhammad Iqbal, Ahmad Saeed, Ghulam Hafeez, Athar Waseem, Fahad R. Albogamy, Zahid Ullah

**Affiliations:** 1 Department of Electrical Engineering, FET, International Islamic University, Islamabad 44000, Pakistan; ebrahim.phdee84@iiu.edu.pk (E.S.); adnan.umar@iiu.edu.pk (A.U.K.); ahmedsaeed771@gmail.com (A.S.); athar.waseem@iiu.edu.pk (A.W.); 2 Research and Innovation Centre of Excellence (KIOS CoE), University of Cyprus, P.O. Box 20537, Nicosia 1678, Cyprus; muhammad.iqbal@iiu.edu.pk; 3 Centre of Renewable Energy, Government Advance Technical Training Centre, Peshawar 25100, Pakistan; 4 Department of Electrical Engineering, University of Engineering and Technology, Mardan 23200, Pakistan; 5 Computer Sciences Program, Turabah University College, Taif University, P.O. Box 11099, Taif 21944, Saudi Arabia; f.alhammdani@tu.edu.sa; 6 Department of Electrical Engineering, University of Management and Technology Lahore, Sialkot Campus, Sialkot 51310, Pakistan; zahid.ullah@skt.umt.edu.pk

**Keywords:** microgrids, fault-tolerant control, fault diagnosis and estimation, sliding mode observers, current/potential transformer, H∞, PI control, Lyapunov stability, robust control, linear matrix inequalities

## Abstract

This work investigates sensor fault diagnostics and fault-tolerant control for a voltage source converter based microgrid (model) using a sliding-mode observer. It aims to provide a diagnosis of multiple faults (i.e., magnitude, phase, and harmonics) occurring simultaneously or individually in current/potential transformers. A modified algorithm based on convex optimization is used to determine the gains of the sliding-mode observer, which utilizes the feasibility optimization or trace minimization of a Ricatti equation-based modification of H-Infinity (H∞) constrained linear matrix inequalities. The fault and disturbance estimation method is modified and improved with some corrections in previous works. The stability and finite-time reachability of the observers are also presented for the considered faulty and perturbed microgrid system. A proportional-integral (PI) based control is utilized for the conventional regulations required for frequency and voltage sags occurring in a microgrid. However, the same control block features fault-tolerant control (FTC) functionality. It is attained by incorporating a sliding-mode observer to reconstruct the faults of sensors (transformers), which are fed to the control block after correction. Simulation-based analysis is performed by presenting the results of state/output estimation, state/output estimation errors, fault reconstruction, estimated disturbances, and fault-tolerant control performance. Simulations are performed for sinusoidal, constant, linearly increasing, intermittent, sawtooth, and random sort of often occurring sensor faults. However, this paper includes results for the sinusoidal nature voltage/current sensor (transformer) fault and a linearly increasing type of fault, whereas the remaining results are part of the supplementary data file. The comparison analysis is performed in terms of observer gains being estimated by previously used techniques as compared to the proposed modified approach. It also includes the comparison of the voltage-frequency control implemented with and without the incorporation of the used observer based fault estimation and corrections, in the control block. The faults here are considered for voltage/current sensor transformers, but the approach works for a wide range of sensors.

## 1. Introduction

Distributed electrical power generation by various renewable and non-conventional micro-sources provides diverse options for configuring modern electrical grids. A microgrid (MG) consists of a cluster of loads and distributed generators (DG) that operate as a single controllable system [1]. The interconnection of DGs to the utility grid through power electronic converters has raised concerns about safe operation and protection of equipment, as power management is required at micro levels as well as for large unified networks. MGs may operate in both autonomous and grid-connected modes, whereas stability and effective control are issues of concern for both modes of operation of dynamic microgrids [2]. A properly simulated and experimentally tested small-signal linearized MG model is required for reliable testing of the observer-based FTC techniques used in this work. The FTC approach enhances the safety and reliability of the entire system by generating alarms for fault magnitudes within certain thresholds. Moreover, it will maintain the operation close to normal instead of immediate shutdowns and prevent possible losses, along with providing maintenance opportunities by shifting the power switches to redundant generator sources using the hierarchical MG control strategy [3,4]. This work is specifically focused on the FTC of microgrids, particularly with reference to sensor faults, as the control of power systems relies on sensor measurements. If the sensors fail, have bad or broken connectivity, cause communication malfunction, etc., they will not only fail the control mechanism but also the power system. In general terms, the reliability enhancement, local voltage support, correction of voltage/frequency sags, and uninterruptible power supply (UPS) are major issues of focus in this work [5]. The small-signal modeling of MGs can be studied in detail in [4,6,7,8,9,10,11,12,13].

Sliding-mode observer (SMO), is different from ordinary Luenberger observers because of the nonlinear switch discontinuous term, which is properly processed for fault diagnostics (detection, isolation, and estimation). They are used for both linear/nonlinear systems with additive faults and are capable of handling a large class of perturbations, parametric variations, uncertainties, and unmodeled dynamics. Moreover, it provides compensation for observer mismatches while replicating the system, along with ensuring stability and reachability in finite time [14,15].

Walcot and Zak [16] used feedback output error linearly in observers, along with a Lyapunov-based stability analysis. The faults can be determined from the deviation of the system trajectory from the sliding surface, but it cannot determine which sensor or actuator is faulty. The basic requirement of fault detection is to detect faults, along with location identification, as mentioned in [17,18]. The difference between the system and observer outputs generates residual signals, which are processed further with static and dynamic thresholds for better diagnostics, reconfiguration of the system/observer, and fault reconstruction. Residual based methods were reviewed by [19,20]. SMO-based methods have also been proposed by [21,22]. Utkin [23] in his proposed observer used a discontinuous term with a suitably scaled gain, which requires software to solve the synthesis problem.

Edwards [24] extended the work by using both discontinuous and linear terms as feedback in SMO, which is transformed into canonical forms by suitable transformations constrained by conditions on invariant zeros of the system. The methods for computing the required gains of the observer are given, which provides an explicit solution, but all degrees of freedom are not exploited. The work was carried out by [25] using an output term injected with an appropriate gain for fault magnitude determination in the localized sensor/actuator while maintaining the sliding motion. Tan [26] used sliding-mode observer (SMO) with linear matrix inequality (LMI) based convex optimization algorithms to exploit more degrees of freedom for observer gains and establish a relationship between the sub-optimal observer and the linear part of the SMO using the linear quadratic Gaussian (LQG) theory. The paper also presented a modified algorithm for pole placement and clustering to place eigenvalues of the linear SMO part in a certain region to attain improved observer performance. The algorithms also involve some design matrices inherent in procedures that can modify the dynamics of the sliding motion. Tan [27] applied the SMO theory to find additive sensor faults by transforming them suitably to act as actuator faults. This work was further extended by [28] for localized additive sensor/actuator fault reconstruction with additive uncertainty in the system. It uses the minimization of the $L_{2}$ gain of uncertainty and reconstructed fault, scaling of the output injection signal, and application of LMIs for more design freedom. Although the sliding mode is often not retained in most cases in a very ideal sense in the presence of faults, the estimation is shown to be made possible in these works.

Ref. [29] also used the idea of correcting the sensor/actuator faults by using the estimated fault, but the technique could not be termed as strictly active or passive. An observer-based approach for online fault estimation was used to correct the sensor faults but was not active in the sense of updating the control law, which was investigated by Edwards in [30]. Yan [31] imposed some conditions on the fault and disturbance distribution matrices to achieve a specific fault reconstruction. Aldeen [32] estimated the states, faults, and disturbances/unknown inputs for nonlinear systems by designing an SMO.

Dhahri [33] initially presented a fault reconstruction methodology for matched faults and uncertainties, which is not very practical because of the very nature of uncertainties; hence, the method is not valid for a good range of practical systems. They further extended the work by determining the SMO gains by feasibility optimization of LMIs attained through Lyapunov stability criteria. It minimizes the H∞ criteria (ratio of residual to disturbance), and the reduced effect of disturbance on fault reconstruction improves the fault estimation, along with validation of the work for unmatched uncertainties/ faults. This study proved the stability and reachability of the sliding-mode observer and evaluated the reachability time by using a suitable mathematical estimation.

Shi [34] presents H∞ based FTC for actuator and sensor faults occurring in a wind energy system. It considers variable wind speed being modeled with stochastic affine models and uses linear quadratic regulator (LQR)-based state feedback control. This paper discusses various intelligent methods for designing and controlling small-and large-scale MGs operating in parallel or in isolation. The increasing complexity of MG systems with various combinations of active and passive elements requires individual converter units to act in a fault-tolerant manner for better efficiency and reliability in generation, distribution, and transmission [35].

In [36], SMO was applied to sensor faults, that is, for current transformer (C.T) magnitude faults and used an estimated fault for correction of sensor (C.T) fault. It also uses feedback pole placement control to track the real and complex powers, and in this way, a compensating sensor (C.T) fault without even replacement or alarm, which can be better termed as a method to design a software-based sensor. An adaptive-fuzzy-PID robust sliding control is proposed for an uncertain class of nonlinear systems, particularly focusing on aircraft flight control, is referred to in [37].

The work in [38] designed a sliding control with adaptive gains and an integral surface for robustness against actuator faults of a wind turbine system to maintain the rotor speed, with simulation results. Recent approaches are moving towards consensus/distributed controls of MGs along with trending applications of fuzzy control. A distributed adaptive fuzzy-based control is presented for large-scale systems with sensor and actuator faults [39].

Kangdi Lu [40] proposed robust PI control for load-frequency control (LFC) of multi area interconnected scenerio, where its parameters are estimated by constrained population extremal optimization. It uses H∞ determined from LMIs as one constraint whereas the integral based time absolute error as another constraint. The papers showed effectiveness of scheme by its comparison with other PI control methods and an optimized model predictive control (MPC). Kang-Di Lu [41] developed adaptive, resilient event triggered PI-based LFC which works with energy constraint. Lyapunov theory to derive criterion of stability which is tested for various case studies, and is proved to be defeating denial of service attacks and reducing the communication burdens.

Damiano Rotando [42] developed LMI-based observer/controller to be employed for dynamic cost estimation and control for a saturated-actuator class of non-linear systems to maintain the cost below an upper bound. The scheme is tested by simulation results on rotational single-arm-inverted pendulum.

This study focuses on designing a sensor FTC mechanism applicable at both the primary and secondary levels with reference to hierarchical MG control [3] strategy. The FTC strategy uses the robust sliding-mode observer (RSMO) theory to be utilized for the detection and estimation of sensor faults of a distributed MG unit operating in autonomous mode of operation, using a reliably simulated and experimentally verified mathematical model of the MG. The considered system has one current transformer (C.T) and potential transformer (P.T), but it is easily scalable for multiple and redundant sensor sources (transformers), which can be utilized for enhanced security and reliability. The online fault estimation-based fault tolerance approach is quite general and can be extended to many types of systems by choosing the suitable observer/controller parameters discussed in this paper. The main contributions of this study are:.

- We have presented an improved method, as it is capable of dealing with either one or both faulty sensors (transformers) with sinusoidal additive faults (composite of magnitude, phase, and harmonics). Earlier work was performed only for magnitude faults in the current transformer in [36].
- Algorithm for determination of SMO gains is modified and improved by using Ricatti-equation-based modification of H∞ enhanced LMIs, i.e., the combination of approaches represented by Edwards [26,33], which enhances robustness of SMO in fault diagnosis and reconstruction. The method for the mentioned approach is discussed in Theorem 2. The comparisons for results produced with SMO gains determined by proposed technique to those determined by earlier/base works (being used in this study), are also presented.
- Fault estimation procedure is modified and contains some factors which were mistakenly missing in reduced order state error equations of earlier works such as [30]. The method also incorporates numerical solution of the DE for reduced order state error estimation. The mentioned approach is worked in Corollary 1.
- Disturbances being unknown inputs are also accurately estimated for all fault types considered in the system. The modified fault estimation method gives very accurate disturbance/unknown inputs estimation as shown in Corollary 1.
- Lyapunov stability analysis of SMO with considered additive faults and disturbances for the considered microgrid system is presented, as shown in Proposition 1.
- Reachability of sliding mode in finite time is proved along with determination of reachability time, for the considered faulty and perturbed microgrid system, as shown in Theorem 3.
- Along with voltage-frequency regulation using PI control and SVPWM in earlier studies, FTC is an additional feature in the same control mechanism by using the corrections in faulty sensor outputs achieved through the estimated faults and disturbances by using SMO.
- The system is simulated for various fault types of practical importance particularly with reference to C.T/P.T and generally for any type of sensors, i.e., sinusoidal, linearly increasing, constant, square pulse, and random type faults with additive sinusoidal harmonics in the form of disturbances.

**Figure 1:**
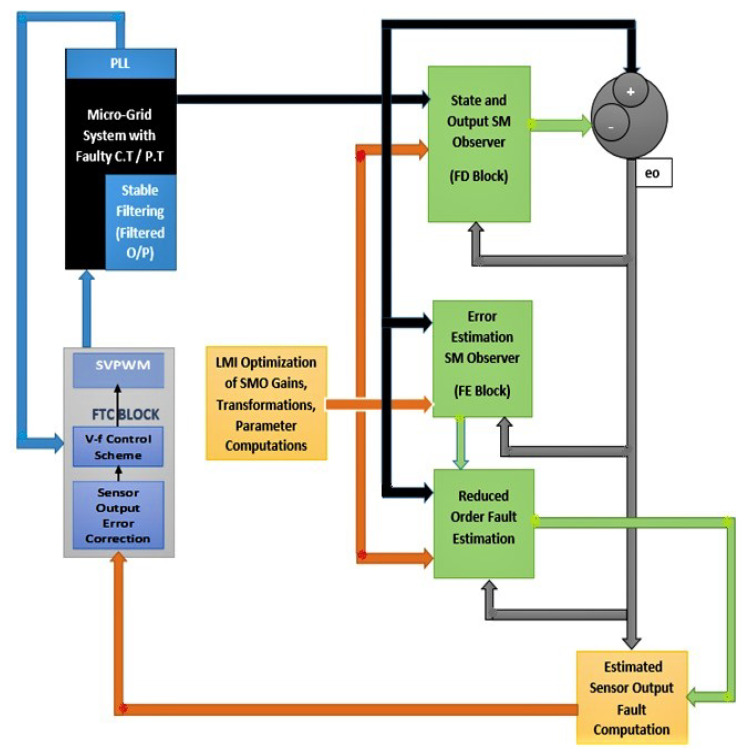
Sensor (C.T/P.T) fault diagnosis and fault-tolerant control approach.

This paper is organized with an introduction, literature review, and brief discussion of the proposed scheme in Section 1, current/voltage transformer faults model and MG system model in Section 2, preliminaries of SMO based detection and estimation theory in Section 3, determination of H∞ optimized SMO gains in Section 4, reduced-order sliding motion dynamics for fault estimation along with stability and reachability proofs in Section 5, voltage-frequency regulation enhanced with FTC in Section 6, results and discussions on various fault cases in Section 7, and concluding remarks and some possible future directions in Section 8. An Appendix A section is also added after the mention of funding and references. A simplified block diagram illustration of this study is presented in Figure 1.

## 2. System Model

### 2.1. Modelling of Current and Voltage Transformer Faults

The actual problem in C.T/P.T is saturation, which causes an increased core magnetization current and reduces secondary current compared to that required by relays and switches. The CT saturation on the primary side is caused by DC offset of the fault current preceding to the additional effects caused by the remnant flux of fault. The relay coils and even wires causing the cumulative impedance burden of the secondary side also plays its role in CT saturation. Hence, protection schemes require several C.Ts connected in parallel [43,44,45]. The inaccuracies of P.T/C.T, their output will result in incorrect signals generated by space-vector-pulse-width-modulation (SVPWM) for voltage source converter (VSC). The VSC will generate consequently the inaccurate voltages/currents, which will not track the required reactive and active powers. The errors of P.T/C.T are sinusoidal in nature inclusive of magnitude, harmonics and phase, whereas the previous studies have considered only magnitude faults for the sake of simplicity [36]. The additive faults/disturbances are considered in the system, such that the sensor output is added up with different sinusoidal faults/disturbances, which compositely form the complete mathematical model of the faults actually occurring in the C.Ts/P.Ts.

(1) $$f\left(t\right)+\xi \left(t\right)=f_{o}*sin\left(\omega_{1}t+\phi_{1}\right)+\xi_{o}*sin\left(\omega_{2}t+\phi_{2}\right)$$

### 2.2. Mathematical Model of Microgrid System

A small-signal MG model is used in this study to determine the workability of the proposed FTC scheme. The MG model is properly simulated, experimentally verified, and able to be used as a block in large grid networks [6].

**Figure 2:**
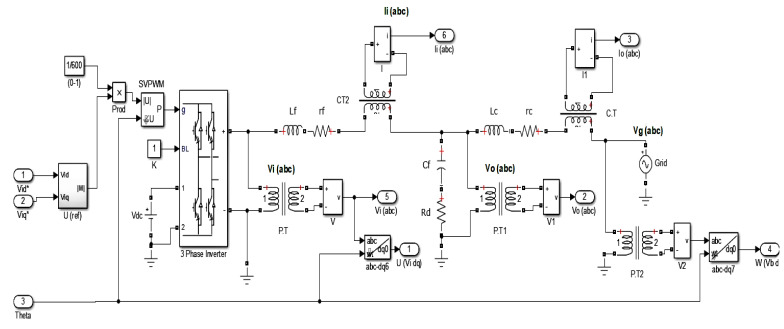
SLD of microgrid.

The mathematical model of a VSC-based microgrid includes non-linear equations; however, its linearized version at the operating point is used in this study. The microgrid model, as proposed by [6] includes inverter equations, voltage source representing grid, LCL filter, PLL, current controller, droop control equations, voltage controller, SVPWM, and reactive and real power calculations, all connected in a cascade to give a non-linear model that needs linearization at the operating point. The LCL filters are connected as passive filters with the VSI, to manage the voltage/current spikes. The line/stray inductances, capacitances, and resistances are also modeled as being considered with LCL filters, i.e., $r_{c}$ and $r_{f}$ are the inductive parasitic resistances, whereas the damping resistor $R_{d}$ is serially connected to the filter capacitor. However, ESR of the capacitor is not considered explicitly instead its lumped into $R_{d}$. The C.T/P.T are mounted on LCL filter to read the instantaneous voltage/current readings, which are used to determine the instantaneous values of reactive and real $\left(Q_{c},P_{r}\right)$ powers. The detailed inverter model as provided in Simulink is considered in this work, i.e., without any major erroneous behavior, it is assumed that the voltage being commanded appears at the input of the filter inductor i.e., $V_{idq}*=V_{idq}$. The losses in the diodes and IGBTs are neglected. The LCL part of the system modeled with KVL/KCL along with average VSI model and grid source taken together is considered as the required part of the mathematical model to design the SMO. Therefore, if there are no sensor faults in (P.T/C.T) of the system, the actual system states/outputs and those of model are in compromise, showing no difference. The considered MG system model is a continuous time LTI system, which follows the separable principle, i.e., the observer and controller can work in combination, and the control action can be performed on the the unknown system states being determined by an observer. The Simulink based model is shown in Figure 2.

**Definition** **1** (Principle of Separation of Estimation and Control). *Considering deterministic linear systems, if state observer based estimated states are used to design a state feedback control for a LTI system, then the observer and feedback controller in combination are stable.*

The linearized state space MG model is given as:

$\left[\begin{array}{c} {\dot{i}}_{i}\\ {\dot{i}}_{o}\\ {\dot{v}}_{o} \end{array}\right]=\left[\begin{array}{ccc} -r_{f}/L_{f} & 0 & -1/L_{f}\\ 0 & r_{c}/L_{c} & 1/L_{c}\\ 1/C_{f}-r_{f}R_{d}/L_{f} & -(1/C_{f}-r_{c}R_{d}/L_{c}) & -(R_{d}/L_{f}+R_{d}/L_{c}) \end{array}\right]\left[\begin{array}{c} i_{i}\\ i_{o}\\ v_{o} \end{array}\right]$

(2) $$+\left[\begin{array}{c} 1/L_{f}\\ 0\\ 1/L_{f} \end{array}\right]v_{i}+\left[\begin{array}{c} 0\\ -1/L_{c}\\ R_{d}/L_{c} \end{array}\right]v_{g}$$

The simulink based microgrid schemetic diagram (SLD) is given in Figure 2. The abc-dq0 transformation is a combination of the Park and Clark transformation for three-phase current/voltage, which is defined for any signal *s*(*t*) as

(3) $$\left[\begin{array}{c} s_{d}\\ s_{q}\\ s_{0} \end{array}\right]≜\sqrt{2/3}\left[\begin{array}{ccc} cos(\theta ) & cos(\theta -\frac{2\pi }{3}) & cos(\theta +\frac{2\pi }{3})\\ -sin(\theta ) & -sin(\theta -\frac{2\pi }{3}) & -sin(\theta +\frac{2\pi }{3})\\ \sqrt{2}/2 & \sqrt{2}/2 & \sqrt{2}/2 \end{array}\right]\left[\begin{array}{c} s_{a}\\ s_{b}\\ s_{c} \end{array}\right]$$

The frequency of the system is determined using PLL while working with dq frame voltages. The grid side angle of voltage is measured by PLL in the considered system, which is then used for all abc-dq/ dq-abc transformations, needed by the system. The *dq* transformed system model in Equation (2) is

$$A_{s}=\left[\begin{array}{cccccc} \frac{-r_{f}}{L_{f}} & W_{pLL} & 0 & 0 & \frac{-1}{L_{f}} & 0\\ w_{pLL} & \frac{r_{f}}{L_{f}} & 0 & 0 & 0 & \frac{-1}{L_{f}}\\ 0 & 0 & \frac{-r_{c}}{L_{c}} & w_{pLL} & \frac{1}{L_{c}} & 0\\ 0 & 0 & -w_{pLL} & \frac{-r_{c}}{L_{c}} & 0 & \frac{1}{L_{c}}\\ \frac{1}{C_{f}}-\frac{r_{f}R_{d}}{L_{f}} & w_{pLL}R_{d} & \frac{-1}{C_{f}}+\frac{r_{c}R_{d}}{L_{c}} & -w_{pLL}R_{d} & -(w_{pLL}+\frac{R_{d}}{L_{f}}+\frac{R_{d}}{L_{c}}) & 0\\ -w_{pLL}R_{d} & \frac{1}{C_{f}}-\frac{r_{f}R_{d}}{L_{f}} & w_{pLL}R_{d} & \frac{-1}{C_{f}}+\frac{r_{c}R_{d}}{L_{c}} & 0 & -(w_{pLL}+\frac{R_{d}}{L_{f}}+\frac{R_{d}}{L_{c}}) \end{array}\right]$$

$$B_{s}=\left[\begin{array}{cc} 1/L_{f} & 0\\ 0 & 1/L_{f}\\ 0 & 0\\ 0 & 0\\ R_{d}/L_{f} & 0\\ 0 & R_{d}/L_{f} \end{array}\right],B_{g}=\left[\begin{array}{cc} 0 & 0\\ 0 & 0\\ -1/L_{c} & 0\\ 0 & -1/L_{c}\\ R_{d}/L_{c} & 0\\ 0 & R_{d}/L_{c} \end{array}\right],C_{s}=\left[\begin{array}{cccccc} 0 & 0 & 1 & 0 & 0 & 0\\ 0 & 0 & 0 & 1 & 0 & 0\\ 0 & 0 & 0 & 0 & 1 & 0\\ 0 & 0 & 0 & 0 & 0 & 1 \end{array}\right],E_{s}=D_{s}\left[\begin{array}{cccc} 1 & 0 & 0 & 0\\ 0 & 1 & 0 & 0\\ 0 & 0 & 1 & 0\\ 0 & 0 & 0 & 1 \end{array}\right]$$

$$x_{s}={\left[I_{id},I_{iq},I_{od},I_{oq},V_{od},V_{oq}\right]}^{T},w={\left[V_{gd},V_{gq}\right]}^{T},u={\left[V_{id},V_{iq}\right]}^{T},\xi ={\left[\xi_{i_{d}},\xi_{i_{q}},\xi_{v_{d}},\xi_{i_{d}}\right]}^{T},f={\left[f_{i_{d}},f_{i_{q}},f_{v_{d}},f_{v_{q}}\right]}^{T},$$

The dq-axis output current and voltage measurements are used to determine the instantaneous reactive power (*Q*${}_{c}$) and active power ($P_{r}$) generated by the inverter.

(4) $$P_{r}=3/2*\left(V_{oq}I_{oq}+V_{od}I_{od}\right)$$

(5) $$Q_{c}=3/2*\left(V_{oq}I_{od}–-V_{od}I_{oq}\right)$$

where $I_{od}$, $I_{oq}$, $V_{od}$, $V_{oq}$ are the dq and components of sensor (P.T/C.T) output currents/voltages. Instantaneous powers are low pass filtered using the corner frequency $\omega_{c}$ to obtain the output power filtered with high frequency effects. The single line diagram (SLD) of the considered VSI-based MG is given Figure 2.

The generalized state-space model of system is:

(6) $$\dot{x_{s}}=A_{s}x_{s}+B_{s}u+B_{g}w$$

(7) $$y_{s}=C_{s}x_{s}+E_{s}f+D_{s}\xi$$

In general form, the dimensions of vectors are $x_{s}\in R^{n*1}$, $x_{h}\in R^{p*1}$, $w\in R^{m*1}$, $u\in R^{m*1}$, $f\in R^{q*1}$, $\xi \in R^{q*1}$, $y_{s}\in R^{p*1}$, whereas the dimensions of system matrix, matrix of grid side dynamics, actuator matrix, and output matrix respectively, are $A_{s}\in R^{n*n}$, $B_{g}\in R^{n*m}$, $B_{s}\in R^{n*m}$, and $C_{s}\in R^{p*n}$, i.e., $\left[0_{p*(n-p)},I_{p*p}\right]$; whereas dimensions of disturbance and fault distribution matrices with full column and row rank are $D_{s}\in R^{q*q}=I_{q*q}$, $E_{s}\in R^{q*q}=I_{q*q}$ respectively, where n≥p≥q.

The Euclidean norm of any vector y is defined by

$$∥y∥≜\sqrt{y^{T}y}$$

The boundedness of the disturbance and fault magnitudes in terms of the Eucledian norm is given by $∥f∥\leq \alpha^{\prime }$ and $∥\xi ∥<\xi_{o}$, which are design requirements, along with the above-mentioned matrix dimensions.

### 2.3. Stable Filtering and Augmented System

A stable filter is used to make the sensor outputs least depending on faults and to magnify the small/ insignificant faults [30]. The system and stable filtered output state vectors are augmented, where the state vector also includes the unscaled actual output variables. In this way, the stable filtered scaled augmented states also provides the isolation of faulty sensors, that is required for proper diagnosis process. A stable filter in some cases may be is a positive definite (PD) scaled identity matrix or a stable matrix with eigenvalues in the left half plane or a high-frequency noise suppressing first-order filter for the output signal in some applications. The state space form of stable filter equation is

(8) $$\dot{x_{h}}=A_{h}y_{s}-A_{h}x_{h}$$

where the stable filter matrix $A_{h}\in R^{p*p}$

(9) $$\dot{x_{h}}=-A_{h}x_{h}+A_{h}C_{s}x_{s}+A_{h}E_{s}f+A_{h}D_{s}\xi$$

The states and stable filtered outputs are augmented for easy handling in a compact form. The system in augmented form is

(10) $$\left[\begin{array}{c} {\dot{x}}_{s}\\ {\dot{x}}_{h} \end{array}\right]=\left[\begin{array}{cc} A_{s} & 0\\ A_{h}C_{s} & -A_{h} \end{array}\right]\left[\begin{array}{c} x_{s}\\ x_{h} \end{array}\right]+\left[\begin{array}{cc} B_{s} & B_{g}\\ 0 & 0 \end{array}\right]\left[\begin{array}{c} u\\ w \end{array}\right]+\left[\begin{array}{c} 0\\ A_{h}E_{s} \end{array}\right]f+\left[\begin{array}{c} 0\\ A_{h}D_{s} \end{array}\right]\xi$$

(11) $${\dot{x}}_{c}=A_{c}x_{c}+B_{c}u_{c}+E_{c}f+D_{c}\xi$$

(12) $$y_{c}=C_{c}x_{c}$$

where $y_{c}\in R^{p*1}$

(13) $$y_{c}=\left[\begin{array}{cc} 0 & I \end{array}\right]\left[\begin{array}{c} x_{s}\\ x_{h} \end{array}\right]$$

where $x_{c}=\left[\begin{array}{c} x_{s}\\ x_{h} \end{array}\right]u_{c}=\left[\begin{array}{c} u\\ w \end{array}\right]$, $A_{c}=\left[\begin{array}{cc} A_{s} & 0\\ A_{h}C_{s} & -A_{h} \end{array}\right]$, $B_{c}=\left[\begin{array}{cc} B_{s} & B_{g}\\ 0 & 0 \end{array}\right]$, $E_{c}=\left[\begin{array}{c} 0\\ A_{h}E_{s} \end{array}\right]$ and $D_{c}=\left[\begin{array}{c} 0\\ A_{h}D_{s} \end{array}\right]$

Dimensions of vectors are $x_{c}\in R^{n_{c}*1}$, $u_{c}\in R^{2m*1}$, $x_{h}\in R^{p*1}$, whereas the dimensions of augmented system matrices are $A_{c}\in R^{n_{c}*n_{c}}$, $B_{c}\in R^{n_{c}*p_{c}}$, $C_{c}\in R^{p_{c}*n_{c}}$, i.e., $\left[0_{p_{c}*\left(n_{c}-p_{c}\right)},I_{p_{c}*p_{c}}\right]$, $A_{h}D_{s}=D_{o}\in R^{q*q}$, $A_{h}E_{s}=E_{o}\in R^{q*q}$, where $D_{c}\in R^{n_{c}*q_{c}}$ and $E_{c}\in R^{n_{c}*q_{c}}$, are disturbance and fault distribution matrices, respectively, in augmented system such that $D_{c}=E_{c}=\left[\begin{array}{c} 0_{n*p}\\ I_{p*p} \end{array}\right]$ and for the MG system under consideration $p_{c}=p=4$, $q_{c}=q=4$, $n_{c}=n+p=10$.

**Remark** **1.** *The invariant zeros of $\left(A_{c},E_{c},C_{c}\right)\subseteq \lambda \left(A_{s}\right)$ if $rank(C_{c}E_{c})\leq q$ as shown in [30], so the system $\left(A_{c},E_{c},C_{c}\right)$ will be minimum phase if the open loop system is stable. In other words, if the system has less inputs than outputs, that is, q≤p, then it is expected that the system will not have any invariant zeros.*

## 3. Fault Diagnosis SMO Filtering

In the literature the phrase ‘fault diagnosis’ refers to three main objectives: detection, isolation, and estimation of faults occurring in the system [46]. The fault detection is often not sufficient in most of the cases, instead it also requires isolation of the fault location along with estimation of faults so that the corrective mechanism can be managed to ensure protection of the systems. These objectives are achieved using the methods which can be categorized into four main classes: model-based, signal-based, observer-based and parameter-estimation-based approaches of fault diagnosis [47,48,49,50]. The observer-based approach for fault detection/ estimation is used in this study, and more particularly it uses SMO for these tasks.

In the next subsection, the basis of the SMOs used for fault detection, isolation, and reconstruction, as shown in earlier works, is briefly discussed.

### 3.1. Fault Detection (FD) SMO Filtering

Fault diagnostics is performed using the SMO, that is, detection and isolation of faults by estimation of states/outputs for the MG application under consideration. In literal sense the isolation functions localization of the faulty sensors of the system; however, the fault estimation SMO is functioning for both the isolation/ estimation of faults. The first-order SMO as proposed by [23,24,30], gives the estimated states of the considered MG system.

(14) $$\dot{x_{o}}=A_{c}x_{o}+B_{c}u_{c}+G_{o}e_{o}+G_{m}\psi$$

where $x_{o}=\left[\begin{array}{c} x_{s}^{*}\\ x_{h}^{*} \end{array}\right]\in R^{n_{c}*1}$ is the estimated state vector which augments estimated system states $x_{s}^{*}\in R^{n*1}$ and estimated stable filtered output states $x_{h}^{*}\in R^{p*1}$. The matrix $G_{o}\in R^{n_{c}*p_{c}}$ is the Luenberger gain of the output estimation error $e_{o}\in R^{p*1}$ term in SMO, which also ensures the stability of the term $\left(A_{o}=A_{c}-G_{o}C_{c}\right)$, whereas $G_{m}\in R^{n_{c}*p_{c}}$ is the gain of the (switching) discontinuous term (ψ) of SMO. Both the gains $G_{o}$ and $G_{m}$ are required to be determined for SMO. The proposed form of the discontinuous term (ψ) is $\left(\psi =-\gamma \frac{P_{o}e_{o}}{∥P_{o}e_{o}∥}\right)$, where the constant gain factor (γ) is being appropriately chosen depending on the application under consideration. The proposed form of the discontinuous term gain $G_{m}=\left[\begin{array}{c} -LT^{T}\\ T^{T} \end{array}\right]$, where the orthogonal matrix $T\in R^{q*q}$ is determined by using QR factorization. The matrices $P_{o}$ and *L* are sub parts of a PD Lyapunov matrix P>0, which is proposed to be of the form $P=\left[\begin{array}{cc} P_{1} & P_{1}L\\ L^{T}P_{1} & T^{T}P_{o}T+L^{T}P_{1}L \end{array}\right]>0$, where the matrices $P\in R^{n_{c}*n_{c}}$, $P_{1}\in R^{n*n}$, $P_{o}\in R^{p*p}$, $T\in R^{p*p}$, $L\in R^{n*p}$ are to be determined ([26]). The Luenberger gain $G_{o}$ is also determined from the Lyapunov matrix *P*, which is explained in detail in the next section.

The output estimated states of the system are given by

(15) $$y_{o}=C_{c}x_{o}$$

The output estimation error of the system is given by

(16) $$e_{o}=y_{o}-y_{c}$$

and

$$A_{c}=\left[\begin{array}{cc} A_{11} & A_{12}\\ A_{21} & A_{22} \end{array}\right],x_{o}=\left[\begin{array}{c} {x_{s}}^{o}\\ {x_{h}}^{o} \end{array}\right]$$

As $e_{o}$ is not the actual output error, instead it is the (scaled) stable filtered output error, so it is suggested empirically to use the form of ψ in Equation (13) instead of its normalized form for the considered MG application.

(17) $$\psi =-\gamma *P_{o}e_{o}$$

**Remark** **2.** *The observer as mentioned in Equation (14) is insensitive to faults (f) completely if:*

1.
$Rank(C_{c}E_{c})=q$

2. *The System triple*$\left(A_{c},E_{c},C_{c}\right)$*has the invariant zeros which lie in Left Half Plane (LHP) [30]*.

### 3.2. State Estimation Error SMO Filtering

The state estimation error is determined by taking the difference of SMO-based estimated states in Equation (14) and system states (being determined) from the mathematical model in (11), being discussed in the previous sections. The difference between Equations (11) and (14) gives the state estimation error.

(18) $$e_{d}=e=x_{o}-x_{c}$$

The state estimation error SMO is

(19) $$\dot{e}=A_{c}e-E_{c}f-D_{c}\xi -G_{o}e_{o}+G_{m}\psi$$

(20) $$\dot{e}=\left(A_{c}-G_{o}C_{c}\right)e-E_{c}f-D_{c}\xi +G_{m}\psi$$

where $A_{o}=A_{c}-G_{o}C_{c}\in R^{n_{c}*n_{c}}$, $e\in R^{n_{c}*1}$

The residual signal (i.e. the output estimation error) in terms of the augmented error vector *e* is given as

$$e_{o}=r\left(t\right)=C_{c}\left(x_{c}-x_{o}\right)=C_{c}e$$

Equation (20) is also a standard form of SMO [23], which is applied to the error system for augmented state error estimation. The state error is further also used for fault/disturbance estimations by attaining the sliding mode, where the state error surface is the considered sliding surface. The next section discusses the stability analysis and determination of gains of the fault detection/estimation SMO observers proposed for the MG system.

## 4. Determination of Sliding Mode Observer Gains through Stability Analysis

A lemma for the existence of the sliding mode is given, before the Lyapunov-based stability analysis of the proposed fault detection/ estimation SMOs for the considered MG system.

**Lemma** **1.** *If σ(e) defines the sliding surface, then for the Lyapunov function*

(21) $$V=e^{T}Pe=e^{T}P^{1/2}P^{1/2}e={∥\sqrt{P}e∥}^{2}$$

*which implies that $\sqrt{V}=∥\sqrt{P}e∥$, $\sigma \left(e\right)=\sqrt{P}e$, and ${∥\sigma ∥}_{2}={∥\sqrt{P}e∥}_{2}$ defines the distance from the sliding surface σ(e)=0. The sliding surface is reached if*

(22) $$\frac{dV}{dt}=\frac{dV}{d\sigma }\frac{d\sigma }{dt}=\sigma^{T}\dot{\sigma }<0$$

*in the neighborhood of surface σ(e)=0 and*

(23) $$\dot{\sigma }=\frac{d\sigma }{de_{o}}\dot{e}$$

**Remark** **3.** *The Lemma discussed above can be studied in detail in [50], as it is the pivotal concept of SMO based FTC techniques used in this work.*

**Proposition** **1.** *If $\left(G_{o}\right)$ is SMO gain for the output error estimation term $\left(e_{o}\right)$, $G_{m}$ the SMO gain of the discontinuous control term (ψ) is proposed to be of the form $G_{m}=\left[\begin{array}{c} -LT^{T}\\ T^{T} \end{array}\right]$, the constant gain (γ) in the (ψ) term is constrained as ($\gamma \geq \eta_{o}-∥E_{o}∥\alpha^{\prime })$ where (η>0) and P is a PD matrix, i.e., (P>0) of the form*

$$P=\left[\begin{array}{cc} P_{1} & P_{1}L\\ L^{T}P_{1} & T^{T}P_{o}T+L^{T}P_{1}L \end{array}\right]>0$$

*which satisfies $\left(PA_{o}+A_{o}^{T}P<0\right)$, then the estimation error e(t) stays bounded and hence asymptotically stable.*

**Proof.**  Let *V* define the Lyapunov function for an augmented error system. The stability of the equilibrium requires the Lyapunov function to be positive definite, and its time derivative to be negative or semi-negative-definite.

(24) $$V\left(e\right)=e^{T}Pe$$

(25) $$\dot{V}\left(e\right)={\dot{e}}^{T}Pe+e^{T}P\dot{e}$$

Using $\dot{e}$ from Equation (19)

(26) $$\dot{V}\left(e\right)=e^{T}{\left(A_{c}-G_{o}C_{c}\right)}^{T}Pe+e^{T}P\left(A_{c}-G_{o}C_{c}\right)e-2e^{T}PE_{c}f-2e^{T}PD_{c}\xi +2e^{T}PG_{m}\psi$$

Using the definition of $G_{m}$ as given in the statement of the proposition, and dropping the negative definite Lyapunov term because $Z=A_{o}^{T}P+PA_{o}<0$, where $A_{o}=A_{c}-G_{o}C_{c}$, the remaining terms are still negative, and the time derivative of the Lyapunov function becomes an inequality, which is always easier to handle in terms of parametric independence.

(27) $$\dot{V}\left(e\right)\leq -2e^{T}PE_{c}f-2e^{T}PD_{c}\xi +2e^{T}PG_{m}\psi$$

Using the definitions of (ψ, $G_{m}$, $PG_{o}=C_{o}^{T}P_{o}$, $PE_{c}=C_{o}^{T}P_{o}E_{o}$, and $e^{T}C_{o}^{T}=e_{o})$,

(28) $$\dot{V}\left(e\right)\leq -2{e_{o}}^{T}C_{o}^{T}P_{o}E_{o}f-2{e_{o}}^{T}C_{o}^{T}P_{o}D_{o}\xi -2\gamma ∥P_{o}e_{o}∥$$

Taking norm and upper bounds $∥f∥<\alpha^{\prime }$, $∥\xi ∥<\xi_{o}$

(29) $$\dot{V}\left(e\right)\leq -2∥e_{o}∥[\gamma ∥P_{o}C_{o}∥+∥P_{o}C_{o}E_{o}∥\alpha^{\prime }+∥P_{o}C_{o}D_{o}∥\xi_{o}]$$

Let if $\gamma ∥P_{o}C_{o}∥+∥P_{o}C_{o}E_{o}∥\alpha^{\prime }+∥P_{o}C_{o}D_{o}∥\xi_{o}\geq \eta_{o}∥P_{o}C_{o}∥$

(30) $$\gamma \geq \eta_{o}-∥E_{o}∥\alpha^{\prime }-∥D_{o}∥\xi_{o}$$

Using the constraint on γ from Equation (30) in Equation (29)

(31) $$\dot{V}\left(e\right)\leq -2\eta_{o}∥e_{o}∥$$

Because the Thau inequality ([51]) for the Lyapunov equation for any PD matrix $P_{o}$ is defined for any vector *x* as [33,50].

$$x^{T}P_{o}^{-1}x\geq \lambda_{min}\left(P_{o}^{-1}\right){∥x∥}_{2}^{2}$$

The Lyapunov function in terms of the error function can be represented in the inequality form as: $\left(e^{T}Ze>\lambda_{min}\left(Z\right)∥e{∥}^{2}\right)$. Using the Thau inequality and Equation (31), the inequality version of Equation (26) becomes:

(32) $$\dot{V}\left(e\right)\leq \lambda_{min}{\left(Z\right)∥e∥}^{2}-2\eta_{o}∥e_{o}∥$$

which proves stability (i.e., negative definiteness) of time derivative of Lyapunov function.

Since $∥x∥>\sqrt{\lambda_{min}\left(P^{-1}\right)}{∥x∥}_{2}$ and can be seen in [33,50].

(33) $$\dot{V}\left(e\right)\leq \lambda_{min}\left(Z\right){∥e∥}^{2}-2\eta_{o}\sqrt{\lambda_{min}\left(P_{o}^{-1}\right)∥\sqrt{P_{o}}e_{o}∥}$$

(34) $$\dot{V}\left(e\right)\leq \lambda_{min}\left(Z\right)\lambda_{min}\left(P^{-1}\right){∥e∥}^{2}-2\eta_{o}\sqrt{\lambda_{min}\left(P_{o}^{-1}\right)∥\sqrt{P_{o}}e_{o}∥}$$

□

The Equation (31) and its representation in terms of (34) show the asymptotic stability of the considered observer system.

**Remark** **4.** *Proposition 1 is analytical proof, particularly for the considered microgrid system with faulty and perturbed sensors using the same steps as performed by Yuri and Edwards in Proposition 3.1 [50], whereas the Thau inequality and observer can be studied in more detail in [51].*

**Theorem** **1.** *Let $Z=A_{o}P+PA_{o}<0$ such that P>0 and the constant gain of the discontinuous switch term γ is constrained by $\gamma \geq \eta_{o}-∥E_{o}∥\alpha^{\prime }$, then the augmented error system (state error and stable filtered output error) dynamics defined by Equation (19) remains bounded, such that the error magnitude remains bounded within the set $X=\{e|∥e∥\leq \frac{2\xi_{o}∥PD_{c}∥}{\lambda_{min}\left(Z\right)}\}$, and the Lyapunov function in vector form gives the constraint in the form of LMI $L_{i1}=\left[\begin{array}{cc} {A_{o}}^{T}P+P\left(A_{o}\right) & PD_{c}\\ -D_{c}^{T}P & 0 \end{array}\right]<0$, where $A_{o}=A_{c}-G_{o}C_{c}$, the LMI is further modified by general algebraic Ricatti equation with additional control parameters to give LMI $L_{i2}=\left[\begin{array}{cc} {A_{c}}^{T}P+PA-C_{c}^{T}{F_{1}}^{-1}C_{c} & Y\\ Y^{T} & {F_{1}}^{-1} \end{array}\right]<0$, and the iterative feasibility optimization or minimization of linear objective (i.e., trace) under LMI constraint $L_{i2}$ gives the SMO gain for output estimation error term to be $G_{o}=P^{-1}C^{T}{F_{1}}^{-1}$, such that Y is constrained to $Y={C_{c}}^{T}{{F_{1}}^{-1}}^{T}>0$, where $\left(F_{1}>0\right)$.*

**Proof.**  Considering the Lyapunov function from Equation (26)

$$\dot{V}\left(e\right)=e^{T}\left(A_{c}-G_{o}C_{c}\right)Pe+e^{T}P\left(A_{c}-G_{o}C_{c}\right)e-2e^{T}PE_{c}f-2e^{T}PD_{c}\xi +2e^{T}PG_{m}\psi$$

Considering switch and fault terms from above equation, i.e., Equation (26)

Let

(35) $$T_{sf}=-2e^{T}PE_{c}f+2e^{T}PG_{m}\psi$$

$T_{sf}=-2e^{T}PE_{c}f+2e^{T}PG_{m}\left(-\gamma \frac{P_{o}e_{o}}{∥P_{o}e_{o}∥}\right)$

(Since $PG_{o}=C_{o}^{T}P_{o}$ and $PE_{c}=C_{o}^{T}P_{o}E_{o}$ )

$T_{sf}=-2e^{T}C_{o}^{T}P_{o}E_{o}f-2\gamma ∥C_{o}P_{o}e_{o}∥$

Taking the norm, we use the Cauchy–Schwartz inequality and bounded fault with $∥f∥\leq \alpha^{\prime }$

$T_{sf}=-2∥e_{o}∥(∥C_{o}P_{o}E_{o}∥\alpha^{\prime }+∥C_{o}P_{o}∥\gamma )$

Because it is desired that the above term stays more negative $T_{sf}<0$, for the negative definiteness of the time derivative of Lyapunov function in Equation (26), which is satisfied if

$∥P_{o}C_{o}E_{o}∥\alpha^{\prime }+∥P_{o}C_{o}∥\gamma >\eta_{o}∥P_{o}c_{o}∥$$\Rightarrow \gamma \geq \eta_{o}-∥E_{o}∥\alpha^{\prime }$□

Furthermore, since $\left(A_{o}=A_{c}-G_{o}C_{c}\right)$, and if $\left(Z=A_{o}P+PA_{o}<0\right)$ where (P>0), so dropping the negative $T_{sf}$ terms, the Lyapunov function is still negative

(36) $$\dot{V}\left(e\right)\leq e^{T}\left(A_{c}-G_{o}C_{c}\right)Pe+e^{T}P\left(A_{c}-G_{o}C_{c}\right)e-2e^{T}PD_{c}\xi$$

To show stability in terms of viable set form

(37) $$\dot{V}\left(e\right)\leq \lambda_{min}{\left(Z\right)∥e∥}^{2}-2\xi_{o}∥PD_{c}∥∥e∥$$

(38) $$\dot{V}\left(e\right)\leq ∥e∥(\lambda_{min}{\left(Z\right)∥e∥}^{2}-2\xi_{o}∥PD_{c}∥)$$

If $\left(∥e{∥}^{2}>\frac{2\xi_{o}∥PD_{c}∥}{\lambda_{min}\left(Z\right)}\right)$, we obtain $\dot{V}<0$, which ensures that the error magnitude remains bounded for the set

$$X=\{e|∥e∥\leq \frac{2\xi_{o}∥PD_{c}∥}{\lambda_{min}\left(Z\right)}\}$$

Using Equation (36) and expressing in terms of the vector quadratic function, LMIs can be determined.

(39) $$\dot{V}\left(e\right)\leq \left[\begin{array}{cc} e^{T} & \xi^{T} \end{array}\right]\left[\begin{array}{cc} A_{o}P+PA_{o} & PD_{c}\\ -D_{c}^{T}P & 0 \end{array}\right]\left[\begin{array}{c} e\\ \xi \end{array}\right]$$

The constraint is true if the matrix in the vector quadratic function is negative definite.

(40) $$L_{i1}=\left[\begin{array}{cc} A_{o}P+PA_{o} & -PD_{c}\\ -D_{c}^{T}P & 0 \end{array}\right]<0$$

The LMI in Equation (40) can be solved for the trace minimization-based algorithm given by [26] to determine the optimized gains of the SMO. However, for the LMIs to be optimized with trace minimization without the H∞ constraint for the determination of traditional SMO gains, it needs some modification. Applying the Schur complement to the LMI in Equation (40)

(41) $${\left(A_{c}-G_{o}C_{c}\right)}^{T}P+P\left(A_{c}-G_{o}C_{c}\right)\leq 0$$

Because the LMI is not feasible in the actual form mentioned by $L_{i1}$ in Equation (40) with a trace-minimization-based optimization algorithm, some modifications are required. By adding/subtracting the term $(C_{c}^{T}{F_{1}}^{-1}C_{c}$) and two more Lyapunov stable terms in above Lyapunov equation to form a balanced an algebraic Ricatti equation, i.e., $\left(YF_{1}Y^{T}\right)$ and (PWP) in the inequality, where $\left(Y=PG_{o}\right)$

(42) $$A_{c}^{T}P+PA_{c}-YC_{c}-{C_{c}}^{T}Y^{T}+{C_{c}}^{T}{F_{1}}^{-1}C_{c}-C_{c}{F_{1}}^{-1}C_{c}+YF_{1}Y^{T}+PW^{-1}P\leq 0$$

(43) $${A_{c}}^{T}P+PA_{c}-C_{c}^{T}\left(Y^{T}-F_{1}^{-1}C_{c}\right)-Y\left(C_{c}-F_{1}Y^{T}\right)-{C_{c}}^{T}{F_{1}}^{-1}C-YF_{1}Y^{T}+PW^{-1}P\leq 0$$

Using/constraining $C_{c}-F_{1}Y^{T}=0$, which is equivalent to $Y^{T}-{F_{1}}^{-1}C_{c}=0$ gives the Luenberger SMO gain

(44) $$G_{o}=P^{-1}C_{c}^{T}{F_{1}}^{-1}$$

Parameters such as the Lyapunov matrix *P* and matrix *F* are missing and need to be determined. Using the constraint $C_{c}-F_{1}Y^{T}=0$, Equation (43) becomes:

(45) $${A_{c}}^{T}P+PA_{c}-C_{c}^{T}{F_{1}}^{-1}C_{c}+PW^{-1}P\leq 0$$

Applying Schur complement on LMI in Equation (45), and the constraint $F_{1}^{-1}C_{c}=Y^{T}$

(46) $$L_{i2}=\left[\begin{array}{cc} A_{c}P+PA_{c}-C_{c}^{T}Y^{T} & P\\ P & -W \end{array}\right]<0$$

**Remark** **5.** *The LMI $L_{i2}$ is optimized by using iterative convex feasibility optimization or alternatively trace minimization, as explained by an algorithm in ([26]), to determine the unknown parameters such as Lyapunov matrix P and matrix F, which are used to determine Luenberger gain $G_{o}$ of SMO. Moreover, the discontinuous term gain, $G_{m}=\left[\begin{array}{c} -LT^{T}\\ T^{T} \end{array}\right]$ as discussed in Proposition 1 can also be determined, as the parameter matrix L is also determined from the part $P_{12}$ of Lyapunov matrix P by the relation $\left(L=P_{11}^{-1}P_{12}\right)$, where the orthogonal matrix T is can be determined by QR factorization.*

The next subsection discusses the H∞ enhanced trace minimization of LMIs to attain the robustness of the SMO for fault estimation with disturbance rejection.

### H*∞* Optimized Robust Sliding Mode Observer Gains (Using LMIs)

H∞ is a robust control criterion that may have several meanings with reference to context; however, in this work, it is desired that the fault detection and estimation be ensured with robustness against disturbances, whereas the H∞ gain will ensure an upper bound of the disturbance voltage/current that will be suppressed to ensure the fault detection/estimation task. The method of incorporating H∞ criteria may also have different approaches; however, this study uses a game-theoretic basis [46]. The criteria are incorporated in the Lyapunov function to derive the LMIs that are convex optimized (using the LMI-optimization toolbox in Matlab) for determination of the SMO gains.

**Definition** **2.** *H∞ based disturbance attenuation problem can be put into formulation as a two-player-zero-sum differential game with disturbance ξ and Luenberger observer gain $G_{o}$ are two players, respectively, where the SMO gains $G_{o},G_{m}$ are required to be designed to minimize the game /cost functional.*

**Lemma** **2.** *The maximum robustness attenuation problem*

(47) $$H\infty =sup_{\left[\xi \neq 0,f=0\right]}\frac{∥r_{H}{∥}_{2,[0,t1]}}{{∥\xi ∥}_{2,[0,t1]}}\leq \mu$$

*for the system given in Equation (7), is satisfied if the cost functional*

(48) $$f_{H}\left(G_{o},G_{m},\xi \right)=\int \left({r_{H}}^{T}r_{H}-\mu \xi^{T}\xi \right)dt,\left(for\right)f=0.$$

*is smaller than or equal to zero for any possible disturbance, where (μ) is the (H∞) parameter and $\left(r_{H}=Hr\left(t\right)=He_{o}=HC_{c}e\right)$ is the output estimation error. The problem is now viewed as a two-player-zero-sum differential game with the above defined cost function in Equation (48), where the minimizing player minimizes the functional through ($G_{o},G_{m}$) and the maximizing player tries to maximize the function through ξ. The details can be seen in Problem 3.1 in [46].*

**Theorem** **2.** *Let $Z=A_{o}P+PA_{o}<0$, where P>0, and if (H>0), then the augmented error system dynamics defined by Equation (19) remain asymptotically stable, and if the Lyapunov function in vector form is enhanced with H∞ constraint, the disturbance attenuation gives the constraint in the form of LMI*

$$L_{i3}=\left[\begin{array}{cc} A_{o}P+PA_{o}+C_{c}^{T}H^{T}HC_{c} & -PD_{c}\\ -D_{c}^{T}P & -\mu I \end{array}\right]<0,$$

*which is further modified by Riccati equation with additional control parameters to give constraint LMI*

$$L_{i4}=\left[\begin{array}{cc} {A_{c}}^{T}P+PA_{c}-C_{c}^{T}Y^{T} & PD_{c}\\ D_{c}^{T}P & -{\mu^{\prime }}^{-1} \end{array}\right]<0,$$

*and the iterative minimization of linear objective (i.e., trace) under LMI constraint $L_{i4}$ gives the Luenberger gain of output error estimation term as $G_{o}=P^{-1}C^{T}{F^{\prime }}^{-1}$, where $Y=PG_{o}>0$ is constrained to $Y={C_{c}}^{T}F>0$ and $W=P\bar{D}P$.*

**Proof.**  Because *V* is a PD Lyapunov function, the negative definiteness of its derivative determines the stability of the system. If the H∞ criterion is used to enhance the robustness of the traditional SMO, the criterion in terms of the norm is defined by

(49) $$∥r_{H}\left(t\right)∥\leq \mu ∥\xi \left(t\right)∥$$

Because the LMI solver constraints for feasible optimization require the residual to be of the form

(50) $$r_{H}\left(t\right)≜Hr\left(t\right)$$

where the output estimation error, i.e., residual is defined by

$$r\left(t\right)=e_{o}=y_{o}-y_{c}=C_{c}e$$

and *H* is a scaling matrix. The Lyapunov function in inequality form in Equation (36) is added with the H∞ criterion according to Lemma 2 as follows:

(51) $$\dot{V}+{r_{H}}^{T}r_{H}-\mu \xi^{T}\xi \leq 0$$

(52) $$\dot{V}\left(e\right)\leq e^{T}\left[\left(A_{c}-G_{o}C_{c}\right)P+P\left(A_{c}-G_{o}C_{c}\right)\right]e+e^{T}C_{c}^{T}H^{T}HC_{c}e-2e^{T}PD_{c}\xi -\mu \xi^{T}\xi$$

where

(53) $$H^{T}H=F^{\prime }=\left[\begin{array}{c} 0\\ F \end{array}\right]$$

The matrix $F\in R^{p*p}$ is a sub part of matrix *H*, which provides a control on the residual signal for stability purposes. However, as discussed above, in the definition of $r_{H}\left(t\right)$, it is suggested by the LMI solver to meet the conditions of feasible optimization.

and if

(54) $$H=\left[\begin{array}{c} H_{1}\\ H_{2} \end{array}\right],{r_{H}}^{T}r_{H}=e^{T}{C_{c}}^{T}H^{T}HC_{c}e=e^{T}C_{c}F^{\prime }C_{c}e$$

where $H\in R^{n_{c}*p_{c}},H_{1}\in R^{n*p},H_{2}=F\in R^{p*p}$

(55) $$\dot{V}\left(e\right)\leq \left[\begin{array}{cc} e^{T} & \xi^{T} \end{array}\right]\left[\begin{array}{cc} A_{o}P+PA_{o}+C_{c}^{T}F^{\prime }C_{a} & -PD_{c}\\ -D_{c}^{T}P & -\mu I \end{array}\right]\left[\begin{array}{c} e\\ \xi \end{array}\right]$$

The LMI, which can be solved for feasibility or trace minimization-based optimization, to determine the optimized parameters of the matrix is

(56) $$L_{i3}=\left[\begin{array}{cc} A_{o}P+PA_{o}+C_{c}^{T}F^{\prime }C_{c} & -PD_{c}\\ -D_{c}^{T}P & -\mu I \end{array}\right]<0$$

The Schur complement can be applied to convert any bi-linearity to linearity. Applying Schur complement on LMI in Equation (56)

(57) $${\left(A_{c}-G_{o}C_{c}\right)}^{T}P+P\left(A_{c}-G_{o}C_{c}\right)+C_{c}^{T}{F^{\prime }}^{-1}C_{c}+PD\left(\mu^{-1}I\right)D^{T}P\leq 0$$

Using $\left(Y=PG_{o}\right)$ and adding subtracting the term $\left(Y{F^{\prime }}^{-1}Y{}^{T}\right)$

(58) $${A_{c}}^{T}P+PA-C_{c}\left(Y-C_{c}^{T}F^{\prime }\right)-Y^{T}\left(C_{c}^{T}-Y{F^{\prime }}^{-1}\right)+Y{F^{\prime }}^{-1}Y^{T}+P\bar{D}P\leq 0$$

where $\bar{D}=D_{c}\left(\mu I\right)D_{c}^{T}$

Setting $\left(Y-C_{c}^{T}F^{\prime }=0\right)$ which is equivalent to $\left(C_{c}^{T}-Y{F^{\prime }}^{-1}=0\right)$ gives

(59) $$G_{o}=P^{-1}C_{c}^{T}{F^{\prime }}^{-1}$$

and the inequality reduces to

(60) $${A_{c}}^{T}P+PA_{c}-Y{F^{\prime }}^{-1}Y^{T}+W\leq 0$$

where $W=P\bar{D}P$

Using Schur complement on inequality and $C^{T}=Y{F^{\prime }}^{-1}$

(61) $$L_{i4}=\left[\begin{array}{cc} {A_{c}}^{T}P+PA_{c}-C^{T}Y^{T} & PD_{c}\\ D_{c}^{T}P & -\mu I \end{array}\right]<0$$

□

**Remark** **6.** *Trace minimization based optimization of Ricatti equation-motivated modification of LMIs, for determination of SMO gains is presented by [26], and H∞ based feasibility optimization of LMIs for determination of SMO gains is used by [33]. However, this work combines the application of both, i.e., to carry out the feasibility or the linear objective (i.e., trace) minimization-based iterative convex optimization of Ricatti equation-based modification of H∞ enhanced LMIs, which can work or may be tested on generally any system, and particularly here being applied on MG under consideration.*

## 5. Reduced Order Sliding Motion

The error system augments state/ stable filtered output error vectors that is,

(62) $$\left[\begin{array}{c} \dot{e_{s}}\\ \dot{e_{o}} \end{array}\right]=\left[\begin{array}{cc} A_{11} & A_{12}\\ A_{21} & A_{22} \end{array}\right]\left[\begin{array}{c} e_{s}\\ e_{o} \end{array}\right]-\left[\begin{array}{c} G_{1}\\ G_{2} \end{array}\right]e_{o}-\left[\begin{array}{c} 0\\ E_{o} \end{array}\right]f-\left[\begin{array}{c} 0\\ D_{o} \end{array}\right]\xi -\left[\begin{array}{c} LT^{T}\\ T^{T} \end{array}\right]\psi$$

where $e=\left[\begin{array}{c} e_{s}\\ e_{o} \end{array}\right];\;G_{o}=\left[\begin{array}{c} G_{1}\\ G_{2} \end{array}\right];\;E_{c}=\left[\begin{array}{c} 0\\ E_{o} \end{array}\right];\;G_{m}=\left[\begin{array}{c} LT^{T}\\ T^{T} \end{array}\right]$

The generalized dimensions of matrices and vectors are $e_{o}\in R^{p*1}$, $e_{s}\in R^{n*1}$, $\xi \in R^{q*1}$, $\psi \in R^{q*1}$, $f\in R^{q*1}$, $A_{11}\in R^{n*n}$, $A_{12}\in R^{n*p}$, $A_{21}\in R^{p*n}$, $A_{22}\in R^{p*p}$, $E_{o}\in R^{q*q}$, $D_{o}\in R^{q*q}$, $G_{o}\in R^{n_{c}*p}$, $G_{1}\in R^{n*p}$, $G_{2}\in R^{p*p}$, $L\in R^{n*p}$, $T\in R^{q*q}$ whereas for the MG system considered $p=4,\;n=6,\;m_{c}=m=2,\;m=2,\;m_{c}=m=2,\;n_{c}=n+p=10,\;p_{c}=p=4,\;q_{c}=q=4$.

A transformation is used to make the state error part of the error system least dependent on faults to retain the sliding motion, along with the attainment of reduced-order sliding motion for fault estimation. Ideally, the fault term in the state error part of the system and the state error term in the output error part should be eliminated; however, such a transformation cannot be designed. Thus, practically and very strictly, the sliding motion may not be retained owing to faults and disturbances, but the transformation not only provides control over the state error part through the gain matrix (L) but also reduces the effect of the state error part in the fault term to a negligible order in terms of magnitude for the considered MG system. However, the reduced-order state estimation error increases with the passage of time, which can be controlled by scalar (δ) and (β) parameters. Applying Transformation

(63) $$T_{L}=\left[\begin{array}{cc} I_{n-p} & L\\ 0 & T \end{array}\right]$$

where its inverse, which explains the form of the discontinuous term gain of the SMO, is given by

(64) $${T_{L}}^{-1}=\left[\begin{array}{cc} I_{n-p} & -LT^{T}\\ 0 & T^{T} \end{array}\right]$$

The transformation obtains the system to attain a reduced-order sliding surface along with fault estimation. The transformation and its inverse also explain the sense of the considered form of the switching term gain $G_{m}$ in the SMO. The orthogonal matrix *T* was determined using the QR transformation method.

(65) $$\bar{A}=T_{L}A{T_{L}}^{-1}=\left[\begin{array}{cc} {\bar{A}}_{11} & {\bar{A}}_{12}\\ {\bar{A}}_{21} & {\bar{A}}_{22} \end{array}\right]$$

where

${\bar{A}}_{11}=A_{11}+LA_{21},{\bar{A}}_{21}=TA_{21}$

${\bar{A}}_{12}=LA_{22})T^{T}-\left(A_{11}+LA_{21}\right)LT^{T}+(A_{12}$

${\bar{A}}_{22}=TA_{22}T^{T}-TA_{21}LT^{T}$

$T_{L}G_{o}=\left[\begin{array}{c} G_{1}+LG_{2}\\ TG_{2} \end{array}\right],T_{L}E_{c}=\left[\begin{array}{c} LE_{o}\\ TE_{o} \end{array}\right],$

(66) $$T_{L}D_{c}=\left[\begin{array}{c} LD_{o}\\ TD_{o} \end{array}\right],T_{L}G_{m}=\left[\begin{array}{c} 0\\ I \end{array}\right],C_{a}T_{L}=\left[\begin{array}{cc} 0 & T \end{array}\right]$$

(67) $$\left[\begin{array}{c} \dot{e_{s}}\\ \dot{e_{o}} \end{array}\right]=\left[\begin{array}{cc} {\bar{A}}_{11} & {\bar{A}}_{12}\\ {\bar{A}}_{21} & {\bar{A}}_{22} \end{array}\right]\left[\begin{array}{c} e_{s}\\ e_{o} \end{array}\right]-\left[\begin{array}{c} G_{1}+LG_{2}\\ TG_{2} \end{array}\right]\left[\begin{array}{c} 0\\ e_{o} \end{array}\right]-\left[\begin{array}{c} LE_{o}\\ TE_{o} \end{array}\right]f-\left[\begin{array}{c} LD_{o}\\ TD_{o} \end{array}\right]\xi -\left[\begin{array}{c} 0\\ I \end{array}\right]\psi$$

(68) $$\dot{e_{s}}={\bar{A}}_{11}e_{s}+{\bar{A}}_{12}e_{o}-\left(G_{1}+LG_{2}\right)e_{o}-LE_{o}f-LD_{o}\xi$$

(69) $$\dot{e_{o}}={\bar{A}}_{21}e_{s}+{\bar{A}}_{22}e_{o}+TG_{2}e_{o}-TE_{o}f-TD_{o}\xi +\psi$$

A reduced-order error system is needed for fault and disturbance estimation if the observer mechanism remains stable along with reachability of the sliding surface in the finite time. The scalar gain (γ) to be used with he output estimation error term $\left(e_{o}\right)$ in the discontinuous control law defined by term (ψ) is also determined from the analytical work of the stability analysis.

### 5.1. Reachability and Stability Analysis

The complete stability analysis of SMOs used for fault detection and estimation is shown in terms of the estimation error as a sliding surface. The error vector comprises of the state estimation error and the stable filtered output estimation error in the augmented vector form. A transformation is used in the above section for reduced-order error dynamics, which is particularly needed for fault and disturbance estimations. Therefore, considering the state error stability from the earlier section in Proposition 1, the Lyapunov stability of only the stable filtered output estimation error part is performed again to show the reachability of the sliding surface and hence ensures the estimation of the fault in finite time. The reachability is directly related to stability, and its analytical work is also used to determine the sliding surface reachability time. Before detailed analytical work, a Lemma for the stability and reachability conditions is provided.

**Lemma** **3.** *If the sliding mode for the surface σ(e) is attained in finite time, that is, σ(e)=0, then the time derivative of the Lyapunov function dV/dt may follow the inequality*

(70) $$\frac{dV}{dt}\leq k{\left(\sqrt{V}\right)}^{z}$$

*must be bounded more strongly, somewhat away from zero. That is, the attraction to the sliding mode will only be asymptotic, if it vanishes too quickly. The solution of inequality in Equation (65) is given by*

(71) $$2\sqrt{V(t)}\leq -kV\left(t\right)+2V_{0}$$

*Because $\sqrt{V}\geq 0$, then the inequality in Equation (71) states that V must reach V=0 in finite time. In addition, because V is proportional to the Euclidean norm of the sliding surface $\sigma (e_{o})$, that is, $∥\sigma (e_{o})∥$, the rate of reaching the sliding surface is bounded away from zero [24,50].*

**Theorem** **3.** *If the augmented state estimation error system defined by Equation (66) is transformed by matrix $T_{L}^{\prime }=\left[\begin{array}{cc} I_{n-p} & L\\ 0 & T \end{array}\right]$ to induce reduced order sliding motion on estimation SMO,*

*and if the constraint on Lyapunov design matrix is,*

*$Q=P_{o}T\left(A_{22}-G_{2}\right)+\left(A_{22}^{T}-G_{2}^{T}\right)T^{T}P_{o}<0)$,*

*and if $\sigma \left(e_{o}\right)=\left\{e_{o}:Ce_{o}=0\right\}$ governs the reduced order sliding motion,*

*such that the magnitudes of fault/ disturbance are bounded, i.e.,*

*$\left(∥f∥<\alpha^{\prime }\right)$ and $\left(∥\xi ∥\leq \xi_{o}\right)$,*

*and if the gain factor (γ) for output error estimation term is bounded by*

*$\gamma \geq ∥T{\bar{A}}_{21}∥∥e_{s}∥-∥TE_{o}∥\alpha^{\prime }-∥TD_{o}∥\xi_{o}+\eta$,*

*then the fault detector/ estimator SMOs utilized for MG system are ensured to be stable in terms of Lyapunov criteria along with the reachability of the sliding motion in the finite time. The finite time required to reach/hit the sliding surface is given by:*

$$T_{R}\leq \sqrt{\frac{e_{o}^{T}P_{o}e_{o}}{\lambda_{min}\left(P_{o}^{-1}\right)}}$$

**Proof.**  Let the Lyapunov function for the error system be $V_{e}$, where the error vector is augmented by the state estimation error and stable filtered output error for the considered system. Thus, the complete Lyapunov function can be represented as:

(72) $$V_{e}=V_{e_{s}}+V_{e_{o}}=e_{s}^{T}P_{1}e_{s}+e_{o}^{T}P_{o}e_{o}$$

Taking the time derivative of the Lyapunov function to prove the stability of the error system based on its negative definiteness.

(73) $$\dot{V_{e}}=\dot{e_{s}^{T}}P_{1}e_{s}+e_{s}^{T}P_{1}\dot{e_{s}}+\dot{e_{o}^{T}}P_{o}e_{o}+e_{o}^{T}P_{o}\dot{e_{o}}$$

Substituting the values for $\dot{e_{s}}$ from Equation (63)

(74) $$\begin{array}{c} \dot{V_{e_{s}}}=e_{s}^{T}{\bar{A}}_{11}^{T}P_{1}e_{s}+e_{o}^{T}{\bar{A}}_{12}^{T}P_{1}e_{s}-f^{T}E_{o}^{T}L^{T}P_{1}e_{s}-\xi^{T}D_{o}^{T}L^{T}P_{1}e_{s}-e_{o}^{T}G_{1}^{T}P_{1}e_{s}-e_{o}^{T}G_{2}^{T}L^{T}P_{1}e_{s}+\\ e_{s}^{T}P_{1}{\bar{A}}_{11}e_{s}+e_{s}^{T}P_{1}{\bar{A}}_{12}e_{o}-e_{s}^{T}P_{1}LE_{o}f-e_{s}^{T}P_{1}LD_{o}\xi -e_{s}^{T}P_{1}G_{1}e_{o}-e_{s}^{T}P_{1}LG_{2}e_{o} \end{array}$$

The stability of the sliding motion in reduced-order is proved by considering the Lyapunov function on the output part of the error vector only.

(75) $$\dot{V_{e_{o}}}={\dot{e_{o}}}^{T}Pe_{o}+e_{o}^{T}P\dot{e_{o}}$$

(76) $$\begin{array}{c} \dot{V_{e_{o}}}=e_{s}^{T}{\bar{A}}_{21}^{T}T^{T}P_{o}e_{o}+e_{o}^{T}{\bar{A}}_{22}^{T}T^{T}P_{o}e_{o}+e_{o}^{T}G_{2}^{T}T^{T}P_{o}e_{o}-f^{T}E_{o}^{T}T^{T}Pe_{o}-\xi^{T}D_{o}TT^{T}P_{o}e_{o}+\\ \psi^{T}P_{o}e_{o}+e_{o}^{T}P_{o}T{\bar{A}}_{22}e_{o}+e_{o}^{T}P_{o}TG_{2}e_{o}+e_{o}^{T}P_{o}TG_{2}e_{o}+e_{o}TP_{o}TE_{o}f-e_{o}^{T}P_{o}TD_{o}\xi +e_{o}^{T}P_{o}\psi \end{array}$$

(77) $$\dot{V_{e_{o}}}=2e_{o}^{T}P_{o}T^{T}{\bar{A}}_{22}e_{o}+2e_{o}^{T}P_{o}T^{T}G_{2}e_{o}+2e_{o}^{T}P_{o}T^{T}G_{2}e_{o}+2e_{o}^{T}P_{o}E_{o}f-2e_{o}^{T}P_{o}D_{o}\xi +2e_{o}^{T}P_{o}\psi$$

(78) $$\begin{array}{c} \dot{V_{e_{o}}}=e_{o}^{T}\left[P_{o}T\left({\bar{A}}_{22}-G_{2}\right)+\left({\bar{A}}_{22}^{T}-G_{2}^{T}\right)T^{T}P_{o}\right]e_{o}+2e_{o}^{T}P_{o}T{\bar{A}}_{21}e_{s}-2e_{o}P_{o}TE_{o}f-\\ 2e_{o}^{T}P_{o}TD_{o}\xi +2e_{o}^{T}P_{o}\psi \end{array}$$

If designed suitably

(79) $$Q=P_{o}T\left(A_{22}-G_{2}\right)+\left(A_{22}^{T}-G_{2}^{T}\right)T^{T}P_{o}<0$$

Dropping the negative definite term, the inequality version of Equation (78) is

(80) $$\dot{V_{e_{o}}}<2e_{o}^{T}P_{o}T{\bar{A}}_{21}e_{s}-2e_{o}P_{o}TE_{o}f-2e_{o}^{T}P_{o}TD_{o}\xi +2e_{o}^{T}P_{o}\psi$$

Taking norm of above equation, and using the Cauchy–Schwartz inequality

(81) $$\dot{V_{e_{o}}}\leq 2∥e_{o}∥∥P_{o}T{\bar{A}}_{21}∥∥e_{s}∥-2∥e_{o}∥∥P_{o}TE_{o}∥∥f∥-2∥e_{o}∥∥P_{o}TD_{o}∥∥\xi ∥-2\gamma ∥P_{o}e_{o}∥$$

(82) $$\dot{V_{e_{o}}}\leq 2∥P_{o}e_{o}∥[∥TA_{21}∥∥e_{s}∥-∥TE_{o}∥∥f∥-∥TD_{o}∥∥\xi_{o}∥-\gamma ]$$

Since Furthermore, since magnitudes of fault and disturbance are bounded: $∥f(t)∥<\alpha^{\prime }$ and $∥\xi (t)∥\leq \xi_{o}<\infty$

(83) $$\dot{V_{e_{o}}}\leq 2∥P_{o}e_{o}∥[∥T{\bar{A}}_{21}∥∥e_{s}∥-∥TE_{o}∥\alpha^{\prime }-∥TD_{o}∥\xi_{o}-\gamma ]$$

The derivative of Lyapunov function is negative definite if

(84) $$\gamma >∥T{\bar{A}}_{21}∥∥e_{s}∥-∥TE_{o}∥\alpha^{\prime }-∥TD_{o}∥\xi_{o}$$

where η is a positive constant that ensures Lyapunov stability in terms of $\dot{V_{e_{o}}}$. Using the bound for γ from Equation (84) in Equation (83),

(85) $$\dot{V_{e_{o}}}\leq -2\eta ∥P_{o}e_{o}∥$$

If *P* is a positive definite matrix, then ${∥x∥}_{2}^{2}>\lambda_{min}\left(P^{-1}\right){∥x∥}_{2}^{2}$ ([51])

$\Rightarrow ∥e_{o}{∥}^{2}=e_{o}^{T}e_{o}=e_{o}^{T}P_{o}^{1/2}P_{o}^{-1}P_{o}^{1/2}e_{o}\geq \lambda_{min}(P_{o}^{-1}{∥e_{o}∥}^{2}$

(86) $$\Rightarrow ∥e_{o}∥\geq \sqrt{\lambda_{min}\left(P_{o}^{-1}\right)}\sqrt{V_{e_{o}}}$$

where $∥P_{o}^{1/2}e_{o}∥=\sqrt{V_{e_{o}}}$.

Using Lemma 3, Equation (85) shows the stability and finite-time reachability of the sliding motion for the sliding-mode observers. The finite-time reachability governs and ensures the real-time operation of the proposed observers for fault detection and estimation, that is, the sliding surface is ensured to be reached/hit in a finite time. Using Equations (79), (80), (84), and (85) in the Lyapunov function in Equation (78), in the inequality form yields:

(87) $$\dot{V_{e_{o}}}\leq \lambda_{min}\left(Q\right)∥e_{o}{∥}^{2}-2\eta ∥P_{o}e_{o}∥$$

Using Equation (86)

(88) $$\dot{V_{e_{o}}}\leq \lambda_{min}\left(Q\right)\lambda_{min}\left(P_{o}^{-1}\right)V_{e_{o}}-2\eta \sqrt{\lambda_{min}\left(P_{o}^{-1}\right)V_{e_{o}}}$$

(89) $$\dot{V_{e_{o}}}\leq \frac{\lambda_{min}\left(Q\right)}{\lambda_{min}\left(P_{o}^{-1}\right)}V_{e_{o}}-2\eta \sqrt{\lambda_{min}\left(P_{o}^{-1}\right)V_{e_{o}}}$$

For the DE of the form ($\dot{x}\left(t\right)\leq -ax\left(t\right)-b\sqrt{x(t)}$) ([33]), the time required for x(t) to move from $x_{0}=x\left(t=0\right)$ to $x_{1}=x\left(t=1\right)$ is

$$T_{R}\leq \frac{2}{a}ln\left(\frac{a\sqrt{x_{o}}+b}{a\sqrt{x_{1}}+b}\right)\leq \frac{2}{b}\left(\sqrt{x_{o}}-\sqrt{x_{1}}\right)$$

The reachability time ($T_{R}$) is given by

(90) $$T_{R}\leq \frac{1}{\eta }\left(\frac{V_{o}\left(e_{o}\left(t_{o}\right)\right)-V_{o}\left(e_{o}\left(t_{o}+T_{R}\right)\right)}{\lambda_{min}\left(P_{o}^{-1}\right)}\right)\leq \sqrt{\frac{e_{o}^{T}P_{o}e_{o}}{\lambda_{min}\left(P_{o}^{-1}\right)}}.$$

where

$$V_{o}\left(e_{o}\left(t_{o}+T_{R}\right)\right)=e_{o}^{T}\left(t_{o}+T_{R}\right)P_{o}e_{o}\left(t_{o}+T_{R}\right)=0$$

□

Because the stability and finite-time reachability of the sliding-mode observers is shown and the gain of the output error injection term is determined, the estimation of faults and disturbances can proceed with the results determined in previous sections.

### 5.2. Reconstructed Fault and Estimated Disturbance

This subsection describes the analytical work of faults and disturbance reconstruction based on the results obtained in the previous sections.

**Corollary** **1.** *Considering the MG system/ observer in Equations (11) and (14) respectively, if the SMO gains are determined by solving the LMI optimization problem described in Theorem 2, the transformed error system is defined by Equations (68) and (69), and if (γ) being constant gain of output error estimation term is constrained to satisfy (84) to ensure the stability of observers, i.e.,*

*$\gamma \geq ∥T{\bar{A}}_{21}∥∥e_{s}∥-∥TE_{o}∥\alpha^{\prime }-∥TD_{o}∥\xi_{o}+\eta$,*

then sliding surface attained in reduced order gives the estimated sensor fault $\left(f^{*}\right)$/ disturbance $\left(\xi^{*}\right)$ to be:

$$f^{*}=\beta E_{o}^{-1}T^{-1}\psi_{eq}$$

$$\xi^{*}=f-f^{*}-E_{o}^{-1}T^{-1}A_{21}e_{s}$$

*where β serves as a scaling constant.*

**Proof.**  When observers enter the sliding mode, that is, the sliding surface is reached, the condition in terms of the error surface ideally is (Ce=0); however, for the considered perturbed and faulty sensor MG systems, the sliding mode/surface is reached only for the output error term $\left(e_{o}=\dot{e_{o}}=0\right)$ and not for the state estimation error term ($e_{s}$). However, if there is no disturbance term, then $\left(e_{s}\to 0;\dot{e_{s}}\to 0\right)$ are also approached in finite time.

(91) $$\dot{e_{s}}={\bar{A}}_{11}e_{s}-LE_{o}f-LD_{o}\xi +T\psi_{eq}$$

(92) $$0={\bar{A}}_{11}e_{s}-TE_{o}f-TD_{o}\xi +T\psi_{eq}$$

From above Equation (92)

(93) $$f=E_{o}^{-1}T^{-1}A_{21}e_{s}-E_{o}^{-1}D_{o}\xi +E_{o}^{-1}T^{-1}\psi_{eq}$$

Using the fault estimate Equation (93) in the state error Equation (94) yields the reduced-order state error equation

(94) $$\dot{e_{s}}=\left({\bar{A}}_{11}-LT^{-1}{\bar{A}}_{21}\right)e_{s}-LT^{-1}\psi_{eq}$$

This DE needs to be solved linearly at every instant of time to be back-substituted in the fault estimation Equation (93) to obtain a closer fault estimate. As evident from Equation (94), the solution to DE also requires a run time evaluation of factor $\left(\psi_{eq}\right)$, because the aforementioned approach is necessary for disturbance estimation. Considering the terms in the fault estimate Equation (93), because the state error term $e_{s}$ in the fault equation cannot be removed or isolated by the transformation $\left(T_{L}\right)$ applied to system (62), but its magnitude becomes insignificant for the considered MG application, whereas the disturbance term in the equation is known to be an undesired and diverging term, the reconstructed fault should be approximated equivalently by (ψ) term only, which is based on the output estimation error $\left(e_{o}\right)$ term, design matrices $\left(E_{o},T\right)$, and gamma (γ) parameter.

So from Equation (93) the reconstructed fault is

(95) $$f^{*}=E_{o}^{-1}T^{-1}\psi_{eq}$$

The equivalent switched control in sliding mode is $\psi_{eq}$ is

(96) $$\psi_{eq}=-\gamma \frac{P_{o}e_{o}}{∥P_{o}e_{o}∥+\delta }$$

where the term $\psi_{eq}$ is meant to maintain the sliding motion in reduced-order motion in the presence of disturbances and faults, and δ is a constant control parameter for this purpose. □

**Remark 7.** *(γ) is constant gain factor of discontinuous control term $\left(\psi_{eq}\right)$ whereas (δ) is its additional constant control parameter. The $\left(\psi_{eq}\right)$ term controls the gradually increasing reduced-order state error $\left(e_{s}\right)$ and the value of (γ) being in a closed loop, which, if not controlled properly, causes a rapidly increasing reduced-order state error $\left(e_{s}\right)$ and hence the total divergence of the whole process. It is empirically suggested that, for the considered MG application, the reconstructed fault is*

(97) $$f^{*}=\beta E_{o}^{-1}T^{-1}\psi_{eq}$$

*where β is a constant magnitude scaling parameter that is required to adjust the attenuation achieved by stable filtering on the output error term.*

*Because $E_{o}=D_{o}\Rightarrow E_{o}^{-1}D_{o}=Identity$, using Equation (97) in Equation (93), the estimated disturbance in the system is given by*

(98) $$\xi =f-f^{*}-E_{o}^{-1}T^{-1}A_{21}e_{s}$$

**Remark** **8.** *The values of parameters (δ, β, γ) are considered in this paper intuitively by hit and trial, however setting the parameters sub optimally can be addressed in future work, which would also provide generic applicability of observer based fault-tolerant approach for many other applications with different fault/disturbance magnitudes and sensitivity requirements.*

**Remark** **9.** *The presence of disturbances and faults result that the sliding motion is not attained in a strict sense; however the effect of disturbances and faults on fault estimation can be minimized using (H∞) norm as presented in Theorem 2 which is ratio of $L_{2}$ norms of residual ($r\left(t\right)=e_{o}\left(t\right)$) and disturbance (ξ), respectively.*

**Remark** **10.** *The state error term $\left(e_{s}\right)$ in output error term and fault term in state error term are undesired in terms of fault estimation. Although the transformation $\left(T_{L}\right)$ cannot ideally achieve the desired elimination of undesired terms, it provides control through the gain matrix (L), which is determined by the LMIs.*

**Remark** **11.** *Moreover the transformation $\left(T_{L}\right)$ reduces the effect of state error part ($e_{s}$) in estimated fault to quite negligible order in terms of magnitude for the considered system which is almost zero in the case of micro-grids. The results were verified through simulations.*

## 6. Fault Tolerant Control

The estimated faults in C.T/P.T are used for corrections in the faulty sensors’ (transformers) readings to avoid the wrong current/voltage readings to calculate the instantaneous powers and correspondingly generation of wrong PWM signals. The corrected dq-currents/voltages of current/potential transformers are given by the vector

(99) $$Y_{sc}=Y_{s}-E_{s}*f^{*}-D_{s}*\xi^{*}$$

The corrected sensor voltages/currents are given to voltage-frequency control block for proper tracking of reference reactive and real powers.

### P−ω and Q−V Control Scheme

The control block mainly utilizes PI control-based current/voltage controllers and uses droop control and a PLL block to regulate real and complex power in relation to frequency and quadrature voltage, respectively. In other words it is said that, the same P−f and Q−V control mechanisms are enhanced as FTC by using observer-based fault estimates for the correction of sensor outputs. The control block is not discussed in detail except for a brief continuation, as it is taken from the work of [6], from which the model of the MG is considered in Section 2. All the system parameters and PI constants are considered to be the same as those given in the aforementioned paper and detailed PhD thesis.

DROOP CONTROL 

The voltage and frequency references for the standalone mode of operation of the microgrids must be internally generated.

(100) $$\omega^{*}=\omega_{n}-mP_{r}$$

(101) $$V^{*}=V_{oqn}-nQ_{c}$$

where $P_{r}=3/2*\left(V_{oq}I_{oq}+V_{od}I_{od}\right)$ and $Q_{c}=3/2*\left(V_{oq}I_{od}˘-V_{od}I_{oq}\right)$ Because the values are accepted directly from the grid and low-pass filtered output power is processed, the high-frequency current and voltage spikes must be neglected.

$P^{\prime }=\frac{\omega_{c,PLL}}{S+\omega_{c,PLL}}P_{r}$, $Q^{\prime }=\frac{\omega_{c,PLL}}{S+\omega_{c,PLL}}Q_{r}$, where $\omega_{c,PLL}=7853.98$ rad/s

(102) $$m=\frac{\omega_{1}-\omega_{2}}{P_{1}-P_{2}};n=\frac{V_{1}-V_{2}}{Q_{1}-Q_{2}}$$

The value of m=n=1/1000 is used for the simulation, which can be varied for different grid ratings and other applications.

PLL BLOCK

The block computes the grid angle by using the phase-locked loop (PLL) at the instant that is then used for all dq conversions, SVPWM block, and other required computations.

(103) $$\dot{{V_{o}}_{df}}=\omega_{c,PLL}{V_{o}}_{d}-\omega_{c,PLL}{V_{o}}_{df}$$

(104) $$\dot{\theta }=\omega_{PLL}\Rightarrow \theta =\int \omega_{PLL}dt$$

For f=60 Hz $\Rightarrow \omega_{PLL}=377$ rad/s and $\omega_{c,PLL}=7853.98$,

(105) $$w_{PLL}=377-{k_{p}}_{PLL}{V_{o}}_{df}-{k_{i}}_{PLL}\int {V_{o}}_{df}dt$$

The values of constants and gains used in simulations are: $k_{p}=0.25,k_{i}=2$, $w_{c}=377$ rad/s.

VOLTAGE CONTROLLER

The PI-based control equations managing integral-based control serve as a voltage controller and generate the corresponding references of the dq components of the current.

(106) $${{i_{i}}_{d}}^{*}=k_{ivd}\int \left(w_{PLL}-w^{*}\right)dt+k_{pvd}\left(w_{PLL}-w^{*}\right)$$

(107) $${{i_{i}}_{q}}^{*}=k_{ivq}\int \left(V_{oq}^{*}-V_{oq}\right)dt+k_{pvq}\left(\left(V{oq}^{*}-V_{oq}\right)\right)$$

The values of constants and gains used in simulations are: $k_{ivd}=0.25,\;k_{ivq}=0.25,\;k_{pvd}=0.5,\;k_{pvq}=0.5$.

CURRENT CONTROLLER

The block uses the desired current values generated by the above block and generates the corresponding voltage values.

(108) $$V_{id}^{*}=-w_{n}L_{f}i_{iq}+k_{icd}\int \left(i_{id}^{*}-i_{id}\right)dt+k_{pcd}\left(i_{id}^{*}-i_{id}\right)$$

(109) $$V_{iq}^{*}=-w_{n}L_{f}i_{id}+k_{icq}\int \left(i_{id}^{*}-i_{id}\right)dt+k_{pcq}\left(i_{iq}^{*}-i_{iq}\right)$$

The values of constants and gains used in simulations are $w_{n}=377,\;k_{pcd}=1$, $k_{pcq}=1$, $k_{icd}=100,\;k_{icq}=100$.

SVPWM CONTROL

The SVPWM control uses a 10,000 Hz frequency for the considered MG operation. The desired voltage values generated by current controllers are transformed back to a three-phase voltage representation and provided to the SVPWM block, which manages the switching sequences and times of inverter switches to generate the desired values of current and voltage by VSC. These current/voltage values correspondingly manage the real power vs. frequency and complex power vs. voltage sags. The converter model used was a detailed model provided in MATLAB (Simulink) instead of the average model.

**Figure 3:**
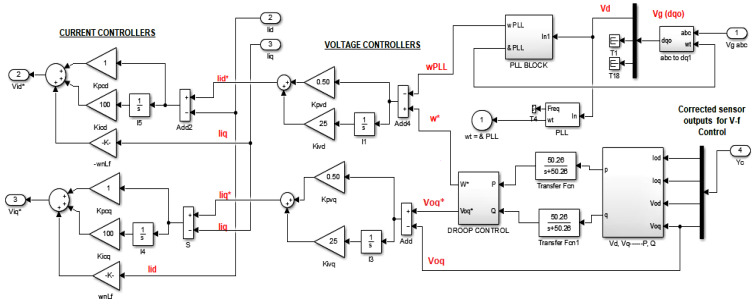
PI and droop control for voltage-frequency regulation.

The complete procedure for the proposed observer-based FTC approach is explained in Algorithm 1.
**Algorithm 1.** Procedure Algorithmine
Inputs: *w*, *u*, ξ, *f*Outputs: Matrices *P*, $G_{m},G_{o}$, $y_{s}$, $x_{s}$, $x_{o}$, *e*, γ, $\Psi_{eq}$, $\xi^{*}$, $f^{*}$, $e_{s}$, $y_{o}$, $e_{o}$while (Simulation Time)START:         **1:** Linearized system model in Equation (2) is d-q-0 transformed using Equation (3);         **2:** Acquisition of grid/converter voltages (*u*, *w*) from microgrid in Figure 2 are given to system model ($\dot{x_{s}}=A_{s}x_{s}+B_{s}u+B_{g}w$), Equation (6)         **3:** Sensor/C.T/P.T faults/disturbances ($f\left(t\right)=f_{o}*sin\left(\omega_{1}t+\phi_{1}\right)$), ($\xi_{o}*sin\left(\omega_{2}t+\phi_{2}\right)$) are generated to be added in system output ($y_{s}=C_{s}x_{s}+E_{s}f+D_{s}\xi$), Equation (7)         **4:** Faulty system output is stable filtered using ($\dot{x_{h}}=A_{h}y_{s}-A_{h}x_{h}$)  Equation (8);         **5:** Augment system states with stable filtered outputs, Equations (10) and (11);         **6:** Determine the SMO gains $G_{o},G_{m})$ using convex (feasibility and trace minimization) optimization performed on system parameters according to methods explained in Theorems 1 and 2;< (i) Perform convex feasibility optimization on the LMI constrained with Lyapunov stability given in Equation (46) (to be used for trace optimization), (ii) Lyapunov stability and H∞ constrained LMI in Equation (56) (to be feasibility optimized), and (iii) Ricatti equation-based modification of H∞ constrained LMI in Equation (61) (to be feasibility optimized), to find the respective optimal gains $G_{o}^{*},G_{m}$. >         **7:** Augmented system state is given to state estimator (SMO) ($\dot{x_{o}}=A_{c}x_{o}+B_{c}u_{c}+G_{o}e_{o}+G_{m}\psi$), Equation (14);         **8:** Difference between system and observer states/outputs ($e_{d}=e=x_{o}-x_{c}$) as defined in Equation (18) gives Augmented state estimation error;         **9:** Augmented system state is given to state error estimator SMO ($\dot{e}=\left(A_{c}-G_{o}C_{c}\right)e-E_{c}f-D_{c}\xi +G_{m}\psi$), Equation (19) or (20);         **10:** Stable filtered output estimation error ($e_{o}=y_{o}-y_{c}$) in Equation (16) is fed to state estimator SMO in step 6 and the state error estimator SMO in step 9 to attain the sliding mode;         **11:** Determine the gains ($\gamma >∥T{\bar{A}}_{21}∥∥e_{s}∥-∥TE_{o}∥\alpha^{\prime }-∥TD_{o}∥\xi_{o}$) and the suitable value of Delta, Equation (84);         **12:** Determine the gain $\psi_{eq}=-\gamma \frac{P_{o}e_{o}}{∥P_{o}e_{o}∥+\delta }$ as in Equation (96);         **13:** Determine the gain reduced order state error $e_{s}$)) by Simulink based numerical solution of ($\dot{e_{s}}=\left({\bar{A}}_{11}-LT^{-1}{\bar{A}}_{21}\right)e_{s}-LT^{-1}\psi_{eq}$) as in Equation (94);         **14:** Compute the estimated fault by ($f^{*}=\beta E_{o}^{-1}T^{-1}\psi_{eq}$), Equation (97)         **15:** Compute the estimated disturbance (unknown input) by ($\xi =f-f^{*}-E_{o}^{-1}T^{-1}A_{21}e_{s}$), Equation (98);         **16:** The erroneous sensor outputs are corrected using estimated faults/disturbances i.e. ($Y_{sc}=Y_{s}-E_{s}*f^{*}-D_{s}*\xi^{*}$), Equation (99).         **17:** The corrected sensor output values are fed to PI/ Droop-based voltage/current/complex power/real power control block, Figure 3;         **18:** Repeat Step 6 and on-wards for the feasibility optimization of H∞ constrained LMI in Equation (56) and the Ricatti equation based modification of H∞ constrained LMI in Equation (61). END (while)

## 7. Results and Discussions

According to the literature [46,47,48], the most commonly occurring faults in sensors are impulsive (incipient), occurring at regular intervals (intermittent), linearly increasing, constant measurement error, and random faults. However, for current and potential transformers in saturation, the faults occurring are mainly sinusoidal magnitude, phase, and harmonic faults. The simulations are performed for state/output estimation, stable filtered state/output estimation, state estimation error, stable filtered output estimation error, state estimation filter error, reduced order state estimation error, disturbance estimation, fault reconstruction, and fault tolerant control (FTC) performance. All these are performed for six fault cases: constant, ramp, sawtooth, square sinusoidal, and random types of additive faults along with additive sinusoidal disturbances of the first, second, and third harmonics. The same types of faults are considered for both voltage and current sensors simultaneously with sinusoidal additive disturbances of different and same frequencies; however, the proposed FD and FTC mechanisms are quite robust against the cross options as well. To avoid unnecessary details and length of paper, the reduced order errors and stable filtered states/outputs are not given for any case, whereas the FTC performance is given for worst-case sinusoidal faults (among the considered ones). The behavior of the system and the results for all simulations were consistent, and no statistical analysis was required for a deterministic system and simulation platform.

After detailed testing of the system, the results are quite good in terms of accuracy, except for the fault and disturbance signals in the near frequency range of 60^o^, which produced scaled current fault estimation in some cases and delayed current fault estimation results in some cases; however, the voltage faults are accurately estimated in all cases. Moreover, there is an occurrence of time delay problem, which needs discrete time compensation for the phase. Some fault estimation errors and less accurate FTC performance of current is due to the very reason. However the problem is not corrected here, instead will be considered for future works.

A Simulink-based detailed three-phased inverter model was considered in the simulation. The grid/ DC source voltage both operate at 600 V. the phase of the grid voltage is used for PLL block and all abc-dq transformations; SVPWM operates at a frequency of 10,000 Hz with sampling time of 0.0001. The discontinuities are caused by greater sampling times, which can be reduced to improve accuracy at the cost of lesser ability of online working due to the increased response time. Because the continuous time simulations move at very low speeds, which are not viable for real-time online performance; therefore, fixed-step solvers are used for simulation in Simulink (MATLAB) with single task handling to avoid complexities with very minor compromises on accuracy.

Regarding some other simulation constants, since the residual magnitude if considered peak to peak is 0.2 for the (*dq*) currents/voltages for the considered time of simulation; whereas the ∥(max(ξ)∥=6.32; so the H∞ norm μ in this case has the numerical value 0.01.

The value of η is a small positive constant considered η=10 to ensure the constraint in inequality (90), whereas the upper bounds for the current and voltage are $\alpha^{\prime }=1A/10V$, respectively. However, for the worst cases and increased magnitudes, it is considered up to 10 A/100 V for I/V, whereas the upper bounds of ξ are normally considered as 0.2 A/2 V for I/V, and for the worst case with increased magnitudes in simulations 3 A/10 V for I/V. The microgrid system model parameters are listed in Table 1, and the controller parameters are listed in Table 2.

### 7.1. Case I—Sinusoidal Faults and Disturbances/Worst Case Scenario (among the Considered Ones)

**Figure 4:**
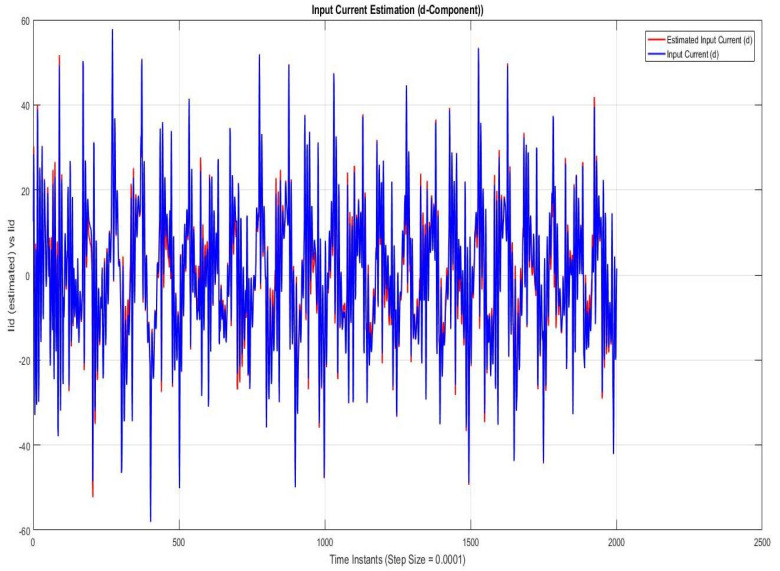
Fault diagnostic observer based input current estimation (d-component).

**Figure 5:**
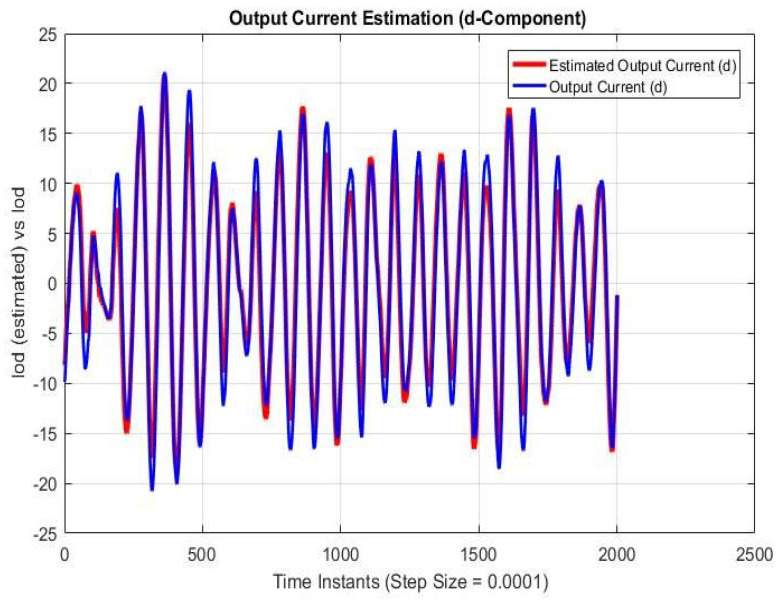
Fault diagnostic observer based output current estimation (d-component).

**Figure 6:**
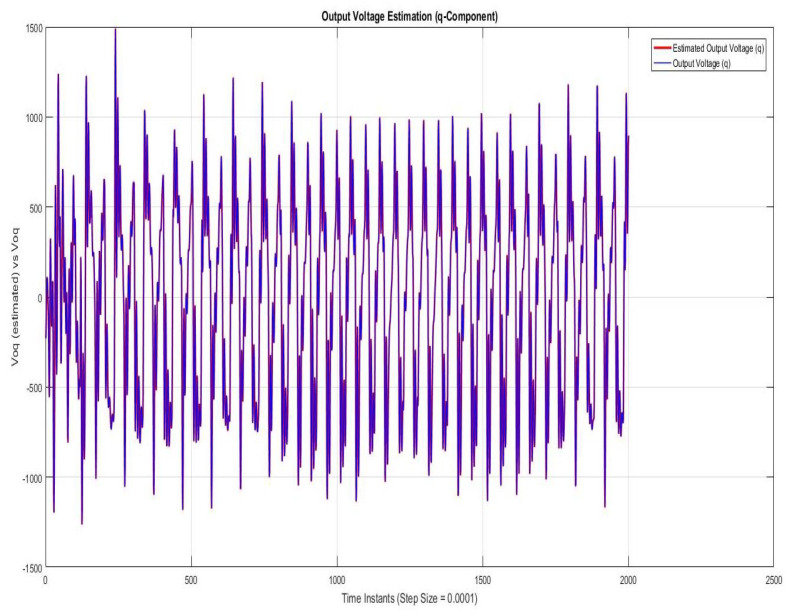
Fault diagnostic observer based state output voltage estimation (q-component).

**Figure 7:**
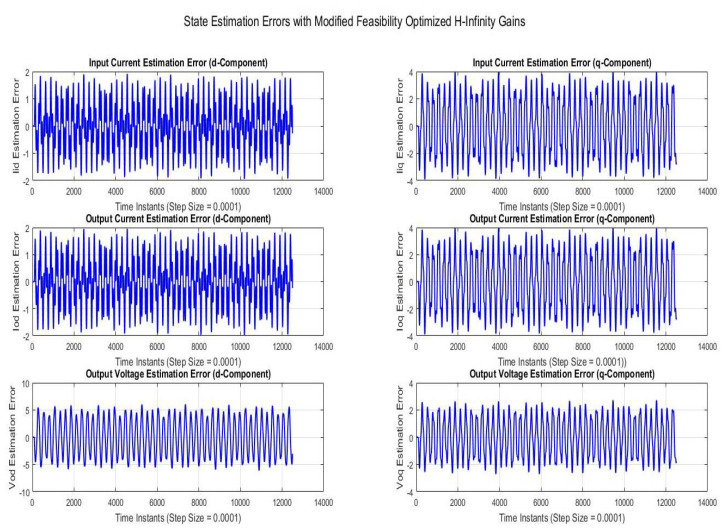
Fault diagnostic observer based state estimation error (dq-components).

**Figure 8:**
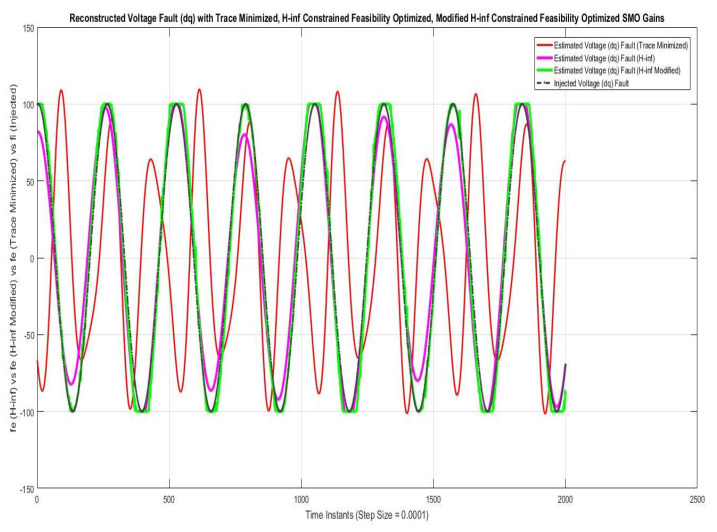
Voltage (dq) fault reconstruction.

**Figure 9:**
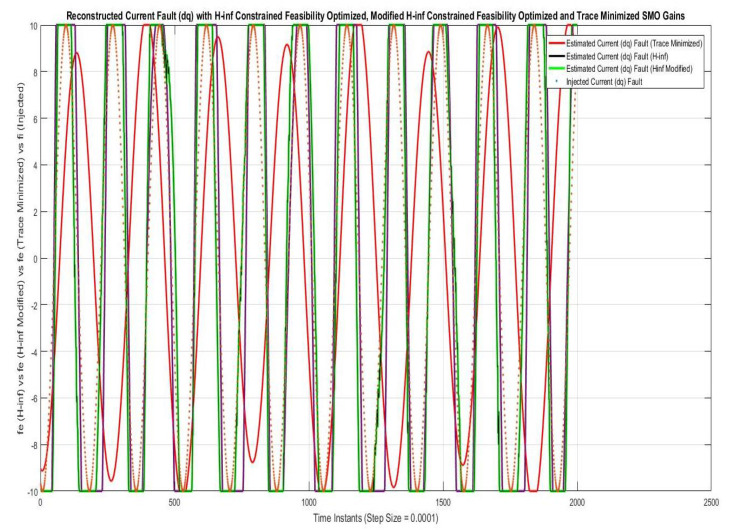
Current (dq) fault reconstruction.

**Figure 10:**
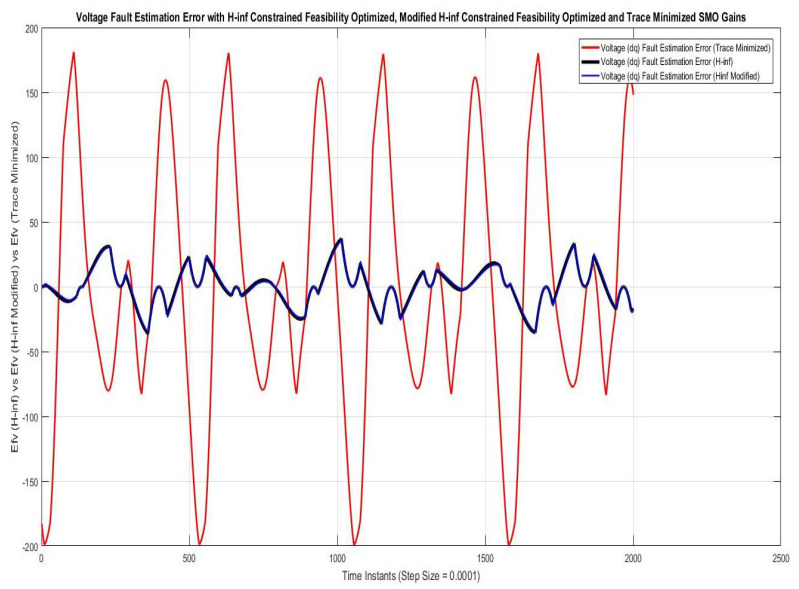
Voltage (dq) fault estimation error.

**Figure 11:**
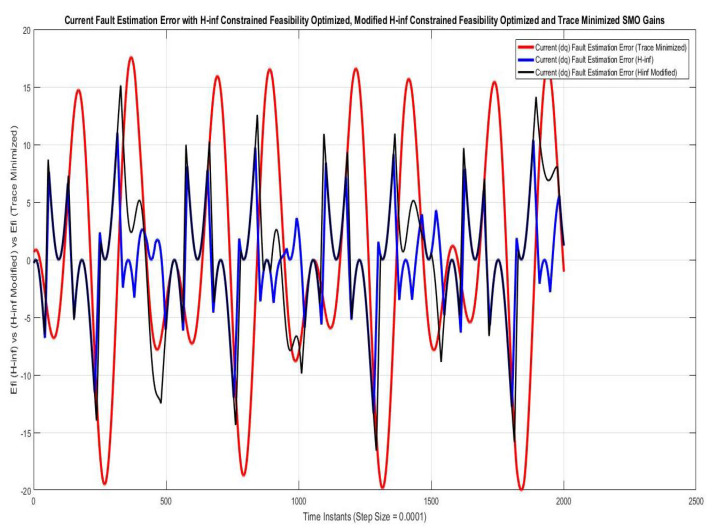
Current (dq) fault estimation error.

**Figure 12:**
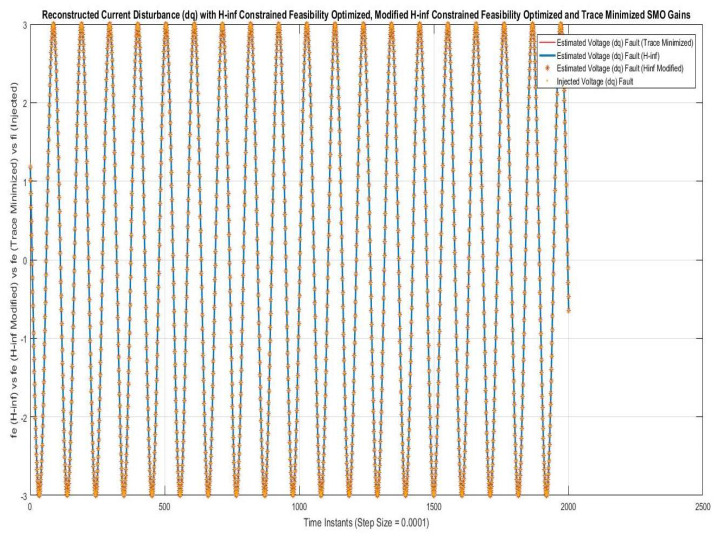
Current (dq) disturbance estimation.

**Figure 13:**
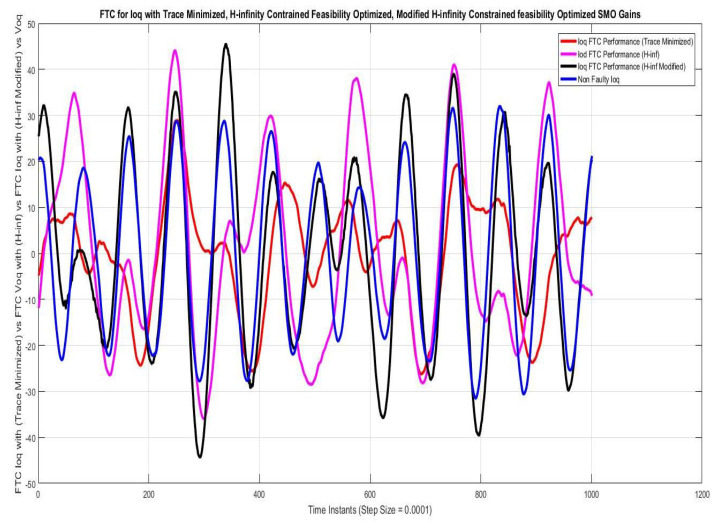
FTC performance for q-component of output current.

**Figure 14:**
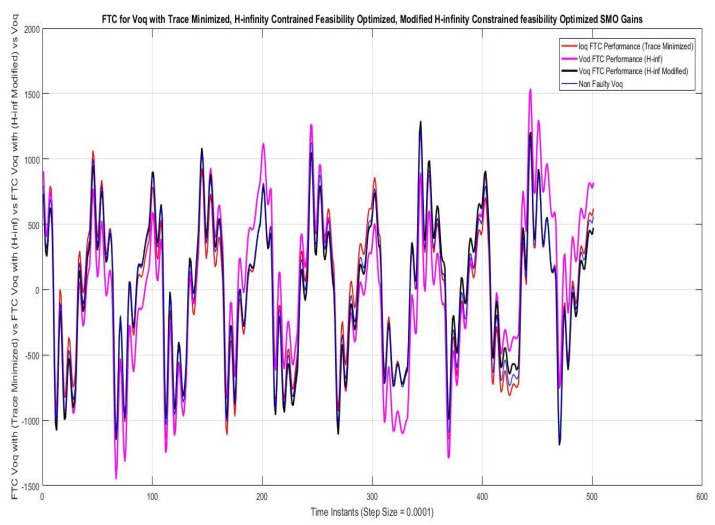
FTC performance for d-component of output voltage.

**Figure 15:**
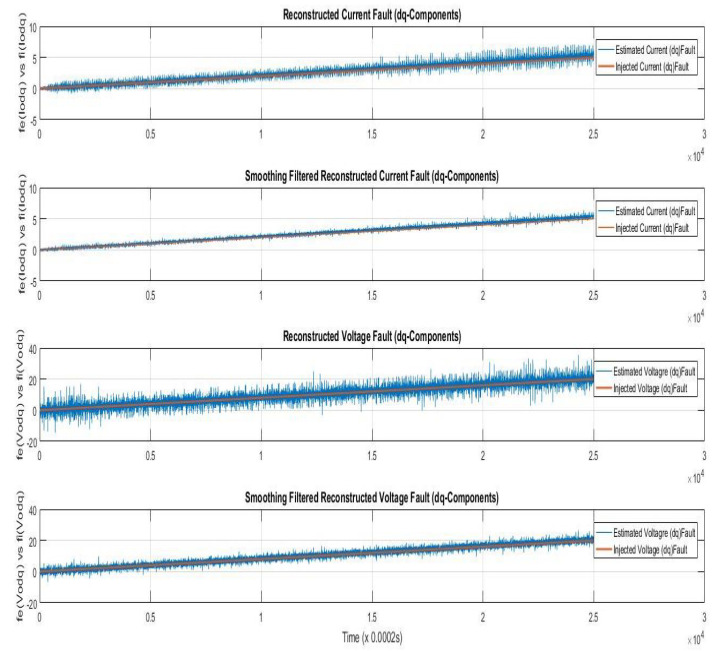
Current (dq) and voltage (dq) fault reconstruction (ramp fault/Case-II).

Simulations were performed for randomly considered different frequencies and phases for both faults and disturbances and quite high fault/disturbance magnitudes, and they provided quite good performance. The voltage and current fault magnitudes are 100 V and 10 A, the frequencies are 120 Hz and 180 Hz, and the phases are $75^{\circ }$ and $240^{\circ }$, respectively, whereas the disturbance magnitudes are 10 V and 3 A, the frequencies are 75 Hz and 300 Hz, and the phases are $45^{\circ }$ and $310^{\circ }$, respectively. The results are shown for the state/output estimation in Figure 4, Figure 5 and Figure 6, the state/output estimation errors in Figure 7, which are performed with modified H∞ constrained SMO gains only. The results in Figure 8 presents voltage fault reconstruction, whereas the current fault reconstruction is presented in Figure 9, voltage fault estimation error in Figure 10, current fault estimation error in Figure 11, current disturbance estimation in Figure 12, FTC performance for q-component of output current in Figure 13, FTC performance for d-component of output voltage in Figure 14 and current and voltage faults reconstruction for ramp/linearly increasing faults in Figure 15. The above mentioned results are compared for SMO gains which optimized for Trace minimization algorithm proposed by Edwards in [25], feasibility optimization of H-infinity enhanced SMO, and Riccatti equation based modification of H-infinity enhanced feasibility optimized gains. Voltage fault reconstruction has a deformation of the lesser grade; however, the current fault reconstruction and fault estimation error increases due to time delay issue, which can be dealt with separately in future works.

An important Remark on Results: The trace minimization of Ricatti-equation-based modification of H∞-enhanced LMI may not be feasible for some forms of LMIs; however the feasibility optimization of the LMI works well nearly in all cases. The minimization of trace is a linear objective for convex optimization, which can work well on some forms of LMIs or some systems depending upon its parameters. However, the trace minimization of Ricatti equation based modification of LMIs not enhanced with H∞ criterion presented by Edwards in [25] also works well nearly in all cases, but its not giving the accuracy of results in the considered microgrid system, which is included in comparisons. This particular aspect still requires much of rigorous testing to comment in a more deterministic way. The feasibility optimized H∞-enhanced-LMIs also have a phase lag effect particularly in current signal, which needs to be compensated, but compensation of phase is not considered in this study.

Figure 4, Figure 5 and Figure 6 are showing the state/output estimation using SMO gains determined by the proposed Riccati equation based modification of H∞ enhanced gains. Instead of all states, the sate estimation performance results are given for estimation of input current (d-component), output current (d-component), Iod, and output voltage )q-component). The results are apparently quite close in terms of estimation except some scaling which can be adjusted further as well.

Figure 7 is showing the state/output estimation error using the modified method of determination of SMO gains. The state estimation error can be considered as a performance index for working of state/output estimator SMO, which also acts as fault detection filter. In the fault magnitude of 10 A/100 V the index stays within a controlled range effectively 1 V/0.5 A, with a negligible small mean value.

Figure 8 and Figure 9 are showing the voltage and current faults estimation/reconstruction using SMOs using the gains optimized with Trace optimization, feasibility optimization of H∞ constrained LMIs, and trace/feasibility optimization of Ricatti equation based modification of H∞ constrained LMIs. The results of the later two as given in proposed technique in Theorem 2 are quite better in terms of accuracy of reconstruction.

Figure 10 and Figure 11 are showing voltage and current fault estimation errors, which can be considered as performance index of working of fault estimations and ultimately the FTC, whose accuracy depends. The results are compared for voltage/current fault estimations performed with SMOs using the gains optimized with Trace optimization, feasibility optimization of H∞ constrained LMIs and trace/feasibility optimization of Ricatti equation based modification of H∞ constrained LMIs. The results of the later two are nearly comparable.

Figure 12 is showing the current disturbances estimation/reconstruction using SMO using gains with the above three methods to lead towards a comparison analysis with the proposed work. The results for all three methods are in compromise and no one method can apparently be said to be better than the others. This accuracy is achieved due to the modified and corrected disturbance estimation procedure mentioned in Corollary 1.

Figure 13 and Figure 14 give the FTC analysis shown for q-components of output voltage. The faulty q-components being corrected by SM observer based fault estimation. The fault estimate and correction using the above mentioned techniques of SMO is taken as comparison, because the accuracy of fault/disturbance estimation ensures the reliable FTC working. The FTC achieved through proposed SMO observer is better as shown by graphical result.

### 7.2. Case II—Ramp Faults and Sinusoidal Disturbances

Simulations are performed in this case for current/voltage injected linearly increasing faults with gradients 4 and 1, respectively, and third-harmonic sinusoidal disturbance, that is, 180 Hz for both V/I. The results for current/voltage fault reconstruction in Figure 15 are given.

## 8. Conclusions and Future Directions

The study uses SMO theory for detection, isolation, estimation, and correction of faults as a scheme of fault-tolerant control, for faults occurring in sensors (current/potential transformers) mounted on microgrid, by using aVSI-based microgrid model. The work is generally applicable for a wide range of sensor/actuator faults and systems. The estimation and correction of faults using SMOs is like providing software based sensors (transformers) replacing the real ones at the time of occurrence of faults. The SMO-based estimated faults are used to correct the faulty readings of the dq-currents/voltages, to be supplied to PI-based conventional voltage-frequency control block, to determine the actual instantaneous values of reactive and real powers, and hence providing the correct SVPWM pulses provided to VSI. The gains of SMOs are determined using convex optimization of Lyapunov stability ensured LMIs. The comparisons for results produced with SMO gains determined by proposed technique to those determined by earlier/base works used in this study, are also presented, which shows the improvements. The comparison of ordinary voltage-frequency control without the proposed SMO-observer-based corrective mechanism is also shown. The state and fault estimation errors are considered as indices to show the effectiveness of the proposed method. The finite-time reachability of detection/estimation SMOs are also presented to show the real-time applicability of the study in VSI-based microgrids. The work sums up various previous works along with modifications in fault estimation and LMI optimization algorithm for determination of SMO gains. The method is transformable to provide fault detection/estimation/tolerance in various types of systems by selecting the suitable parameters.The work is intended to be enhanced for networked microgrids using the approaches of distributed controls while managing the optimal power flow.

## Figures and Tables

**Table 1:** Microgrid system parameters.

Parameter	Value	Parameter	Value
$V_{dc}$	600 V	$V_{g}$	600 V
$L_{f1}$	4.20 mH	$L_{f2}$	4.20 mH
$L_{c1}$	0.50 mH	$L_{c2}$	0.50 mH
$C_{f1}$	15 μF	$C_{f2}$	15 μF
$R_{d1}$	2.025 Ω	$R_{d2}$	2.025 Ω
$r_{f1}$	0.50 Ω	$r_{f2}$	0.50 Ω
$r_{f1}$	0.09 Ω	$r_{f2}$	0.09 Ω
$\omega_{c}$	50.26 rad/s	$\omega_{n}$	377 rad/s
$V_{oqn}$	85 V	m,n	1/1000
$\theta_{grid}$	$60^{\circ }$	$L_{f3}$	4.20 mH
$L_{c3}$	0.50 mH	$C_{f3}$	15 μF
$R_{d3}$	2.025 Ω	$r_{f3}$	0.50 Ω
$r_{f3}$	0.09 Ω	$\omega_{PLL}$	377 rad/s
ine $\omega_{c,PLL}$	7853.98 rad/s		

**Table 2:** Controller gains/parameters.

PI Gains	Parameter	Value
Voltage	$k_{pvd}$, $k_{pvq}$	0.5
Controllers	$k_{ivd}$, $k_{ivq}$	25
Current	$k_{pcd}$, $k_{pcq}$	1
Controllers	$k_{icd}$, $k_{icq}$	100
PLL	$kp_{PLL}$	0.25
Controller	$ki_{PLL}$	2

## Data Availability

The data including Simulink-based simlation files, Matlab based optimization m-files, supplementary result file and some necessarily required documents will be uploaded as the article gets published.

## Nomenclature

Sliding Mode Observer	SMO	Sliding Mode Control	SMC
Robust Sliding Mode Observer	RSMO	Load Frequency control	LFC
Infemum/Supremum	inf/sup	Differential Equation	DE
Fault Detection/Diagnostics	FD	Fault Estimation	FE
Un-interrupted Power Supply	UPS	Voltage Source Converter	VSC
Current/Potential Transformer	C.T/P.T	Phase Locked Loop	PLL
Inductor-Capacitor-Inductor	LCL	Wind Energy System	WES
Microgrid	MG	Linear Quadratic Gaussian	LQG
Linear Quadratic Regulator	LQR	Genetic Algorithm	GA
Particle Swarm Optimization	PSO	Positive Definite	PD
Model Reference Adaptive Control	MRAC	Photo-voltaic	PV
Linear Matrix Inequality	LMI	Linear Parameter Varying	LPV
Model Predictive Control	MPC	Fault-Tolerant Control	FTC
Left/Right Half Plane	LHP/RHP	Proportional-Integral	PI
Proportional-Integral-Differentiator	PID	Distributed Generators	DGs
Phase Locked Loop Frequency	$\omega_{p}LL$	Fault/Disturbance	*f*/ξ
Discontinuous Term	ψ	Discontinuous gain	γ
H-Infinity Coefficient	μ	Fault upper bound	$\alpha^{\prime }$
Disturbance upper bound	$\xi_{o}$	Scaling factor	β
Reachability Time	$T_{R}$	H-Infinity Norm	H∞
Minimum Eigen Value	$\lambda_{min}$	Sliding surface	σ(.)
Space Vector Pulse width Modulation	SVPWM

## Appendix A

### Proof for Proposed Form of Matrix ‘P’

For the form of the Lyapunov matrix *P* as used in [23,26], for the complete Lyapunov function in Equation (73), substituting complete forms from Equations (74) and (77) in (73)

(A1) $$\dot{V_{e}}=\left[\begin{array}{cccc} e_{s}^{T} & e_{o}^{T} & f^{T} & \xi^{T} \end{array}\right]\left[M\right]\left[\begin{array}{c} e_{s}\\ e_{o}\\ f\\ \xi \end{array}\right]$$

$M_{1}=\left[\begin{array}{cccc} {\bar{A_{11}}}^{T}P_{1}+P_{1}\bar{A_{11}} & P_{1}\bar{A_{12}}+\bar{A_{21}}T^{T}P_{o}-P_{1}G_{1}-P_{1}LG_{2} & P_{1}LE_{o} & P_{1}LD_{o}\\ {\bar{A_{12}}}^{T}P_{1}+P_{o}T\bar{A_{21}} & {\bar{A_{22}}}^{T}TP_{o}+P_{o}T\bar{A_{22}}-G_{2}^{T}T^{T}P_{o}-P_{o}TG_{2} & P_{o}TE_{o} & P_{o}TD_{o}\\ E_{o}^{T}L^{T}P_{1} & E_{o}^{T}T^{T}P_{o} & 0 & 0\\ D_{o}^{T}L^{T}P_{1} & D_{o}^{T}T^{T}P_{o} & 0 & \mu I \end{array}\right]$,

and Considering $P=\left[\begin{array}{cc} P_{11} & P_{12}\\ P_{21} & P_{22} \end{array}\right]>0$ (and) $\left[\begin{array}{cc} \bar{A_{11}} & \bar{A_{12}}\\ \bar{A_{21}} & \bar{A_{22}} \end{array}\right]$ in term ${\left(A_{c}-GC_{c}\right)}^{T}P+P\left(A-GC\right)+C_{c}^{T}C_{c}$ and comparing with concerning part of matrix given in vector Lyapunov equation in (104) implies

(A2) $$\bar{P}=\left[\begin{array}{cc} P_{1} & 0\\ 0 & T^{T}PoT \end{array}\right]$$

Because the form of $\bar{P}$ is determined from the $T_{L}$ transformed system, applying the inverse transformation $T_{L}^{-1}\bar{P}T_{L}$ gives

(A3) $$P=\left[\begin{array}{cc} P_{1} & P_{1}L-LP_{o}T\\ 0 & P_{o}+T^{T}P_{1}L \end{array}\right]$$

Applying the transformation ${T_{L}}^{T}PT_{L}$ on $\bar{P}$ gives:

(A4) $$P=\left[\begin{array}{cc} P_{1} & P_{1}L\\ L^{T}P_{1} & T^{T}P_{o}T+L^{T}PL \end{array}\right]>0$$
